# Drivers of wood‐inhabiting fungal diversity in European and Oriental beech forests

**DOI:** 10.1002/ece3.11660

**Published:** 2024-07-03

**Authors:** Giorgi Mamadashvili, Antoine Brin, Maksym Chumak, Valeriia Diedus, Lars Drössler, Bernhard Förster, Kostadin B. Georgiev, Tigran Ghrejyan, Ruslan Hleb, Mark Kalashian, Ivan Kamburov, Gayane Karagyan, Joni Kevlishvili, Zviad Khutsishvili, Laurent Larrieu, Meri Mazmanyan, Peter I. Petrov, Levan Tabunidze, Claus Bässler, Jörg Müller

**Affiliations:** ^1^ Field Station Fabrikschleichach, Department of Animal Ecology and Tropical Biology Biocenter, University of Würzburg Rauhenebrach Germany; ^2^ Sciences and digital department University of Toulouse, Ecole d'Ingénieurs de PURPAN, UMR INRAE‐INPT DYNAFOR Toulouse France; ^3^ Department of Entomology and Biodiversity Preservation Uzhhorod National University Uzhhorod Ukraine; ^4^ State Museum of Natural History, National Academy of Sciences of Ukraine Lviv Ukraine; ^5^ Forestry Research and Competence Center ThüringenForst AöR Gotha Germany; ^6^ Chair for Strategic Landscape Planning and Management Technical University of Munich Freising Germany; ^7^ Hessian State Agency for Nature Conservation, Environment and Geology Hesse Germany; ^8^ Laboratory of Entomology and Soil Zoology Scientific Center of Zoology and Hydroecology NAS RA Yerevan Armenia; ^9^ Forestry laboratory Carpathian Biosphere Reserve Rakhiv Ukraine; ^10^ Strandzha Nature Park Directorate Malko Tarnovo Bulgaria; ^11^ Biodiversity Conservation Center “Nacres” Tbilisi Georgia; ^12^ Université de Toulouse, INRAE, UMR DYNAFOR Castanet‐Tolosan France; ^13^ CNPF‐CRPF Occitanie France; ^14^ University of Forestry Sofia, Field Base Petrohan Barzia Bulgaria; ^15^ Caucasus Nature Fund – CNF Tbilisi Georgia; ^16^ Ecology of Fungi, Bayreuth Center of Ecology and Environmental Research (BayCEER) University of Bayreuth Bayreuth Germany; ^17^ Conservation and Research Department Bavarian Forest National Park Grafenau Germany

**Keywords:** deadwood fungi, *Fagus orientalis*, *Fagus sylvatica*, habitat heterogeneity, Hill numbers, species diversity

## Abstract

The hyperdiverse wood‐inhabiting fungi play a crucial role in the global carbon cycle, but often are threatened by deadwood removal, particularly in temperate forests dominated by European beech (*Fagus sylvatica*) and Oriental beech (*Fagus orientalis*). To study the impact of abiotic drivers, deadwood factors, forest management and biogeographical patterns in forests of both beech species on fungal composition and diversity, we collected 215 deadwood‐drilling samples in 18 forests from France to Armenia and identified fungi by meta‐barcoding. In our analyses, we distinguished the patterns driven by rare, common, and dominant species using Hill numbers. Despite a broad overlap in species, the fungal composition with focus on rare species was determined by *Fagus* species, deadwood type, deadwood diameter, precipitation, temperature, and management status in decreasing order. Shifting the focus on common and dominant species, only *Fagus* species, both climate variables and deadwood type remained. The richness of species within the deadwood objects increased significantly only with decay stage. Gamma diversity in European beech forests was higher than in Oriental beech forests. We revealed the highest gamma diversity for old‐growth forests of European beech when focusing on dominant species. Our results implicate that deadwood retention efforts, focusing on dominant fungi species, critical for the decay process, should be distributed across precipitation and temperature gradients and both *Fagus* species. Strategies focusing on rare species should additionally focus on different diameters and on the conservation of old‐growth forests.

## Graphical abstract

Fungal composition on the single object was low, however, the multiple regression on distance matrices, identified significant environmental variables determining the community composition. For *q* = 0, the Fagus species, the type of deadwood, the diameter, the elevation, and management were identified as significant variables in descending order. With increasing Hill‐numbers only Fagus species, elevation and type of deadwood remained significant determinants for community composition.

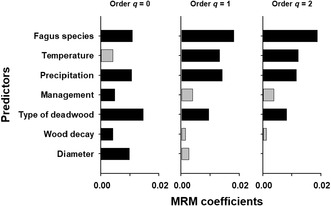

## 1 INTRODUCTION

In the western part of the Palearctic, temperate forests are dominated by two species of *Fagus*: European beech *Fagus sylvatica* in the West and Oriental beech *Fagus orientalis* in the East, *ranging* from eastern Bulgaria to the Hyrcanian forest in Iran. The latter are often Arcto‐tertiary relicts, harboring many endemic plants and animals (Aliev, 2021; Fayvush & Aleksanyan, 2021; Goginashvili & Tvauri, 2013; Mathew et al., 2000). While for European beech the impact of forest management on several taxa has been well studied during the last decades (Brunet et al., 2010; Gossner et al., 2013; Hagge et al., 2019; Morales‐Hidalgo et al., 2015; Ódor et al., 2006), drivers of biodiversity in Oriental beech are not well understood. Most of the studies in European beech forests have identified the loss of old growth trees and the reduction of deadwood amount by forest management as critical for biodiversity (Brunet et al., 2010; Gossner et al., 2013). As a consequence, the amount of deadwood has been selected as one of nine pan European indicators for maintenance, conservation, and appropriate enhancement of the biological diversity in forest ecosystems (Schuck et al., 2015).

Aware of the global responsibility for beech forest ecosystems, conservation strategies have increasingly focused on these ecosystems. Today, European beech forests are well represented in the Natura 2000 network of protected areas (Bohn & Neuhäusl, 2000), while Oriental beech forests of the Caucasus region belong to the Emerald Network of Areas of Special Conservation Interest (Artsivadze et al., 2018; Fayvush et al., 2016). Moreover, several remnants of natural beech forests have received the UNESCO World Heritage designation, including “The Ancient and Primeval Beech Forests of Carpathians and Other Regions of Europe,” “Colchic Rainforests and Wetlands,” and the “Hyrcanian Forest” (UNESCO, 2013, 2019, 2021). Despite the unique fauna and flora of these Tertiary relicts, many managers of Oriental beech forests are often not aware of the key role of deadwood for biodiversity and ecosystem functioning. This became particularly clear when Iran decided to protect the whole Hyrcanian forest and restricted tree removal on deadwood and old growth trees, which would cause a major threat to deadwood organisms (Müller et al., 2016, 2017). This underlines the need to combine findings and research from regions of both beech species. Moreover, in the context of climate change, European forest managers are considering Oriental beech as a potential tree in the future (Mellert & Šeho, 2022). This seems justified because a recent presence of this species in Italy is proved from 45,000‐year‐old DNA pollen samples (Paffetti et al., 2007) and because many saproxylic species specialization to trees is more at the genus than the species level as shown recently for saproxylic beetles (Vogel et al., 2020).

Fungi form a functionally important and hyper‐diverse group in beech forests, acting as mycorrhiza symbionts supporting forest productivity and as key decomposers of litter and deadwood. During deadwood decomposition, many coexisting fungi create habitat heterogeneity and new resources for many other wood inhabiting organisms, and play a crucial role in nutrient cycling processes (Boddy, 2001; Friess et al., 2019; Gessner et al., 2010; Parisi et al., 2018; Valentín et al., 2014). The lignin barrier with the cellulose and hemicelluloses of deadwood, which hinders the polysaccharides from microbial decomposition is only disintegrated and opened for other deadwood dwelling organisms by the help of fungi species and their various secretion of enzymes (Dix & Webster, 1995; Fengel & Wegener, 1983; Floudas et al., 2012; Hoppe et al., 2016; Liers et al., 2006; Stokland et al., 2012).

The drivers of wood‐inhabiting fungi diversity in beech forest depends on the spatial scale. At the local scale of a deadwood object, the tree species, decay stage, type of deadwood and microclimate are important drivers (Baber et al., 2016; Daniel & Nilsson, 1998; Englmeier et al., 2023; Krah et al., 2018; Müller et al., 2020; Purahong et al., 2018; Rajala et al., 2012; Rayner & Boddy, 1988), all affected by local forest management (Abrego, Christensen, et al., 2017; Bässler et al., 2014; Müller, Engel, et al., 2007). At the larger scale, connectivity and macroclimate became more important. For example, Abrego et al. (2015), Abrego, Christensen, et al. (2017) and Heilmann‐Clausen et al. (2014) identified forest connectivity, condition and decay stage of substrates and the climate across European beech forests as the most important factor for fungal species communities in protected areas. Furthermore, Ódor et al. (2006) investigated semi‐natural beech forests in Europe and showed that the diversity of saproxylic organisms is driven mostly by climate and forest management, deadwood volume and habitat fragmentation. Finally, Hagge et al. (2019) showed that the functional diversity of wood‐inhabiting fungi European wide is determined by the latitude, elevation, forest cover, and urbanization.

Despite an increasing interest in beech forests of both *Fagus* species, systematic studies among the entire beech forest belt from France to Caucasus and Iran are widely missing. However, only in such a synopsis it is possible to test the influencing factors of the two beech species in comparison to local factors such as deadwood type, decomposition, diameter, climate, and management for biodiversity. To unify biodiversity research of wood‐inhabiting fungi in European and Oriental beech forests, and to guide future conservation strategies, we conducted a sampling campaign from France in the West to Armenia and Georgia in the East. Using drilling samples and metabarcoding, we aimed at identifying the drivers of fungal communities in *Fagus* forests of both species. In specific, we were interested in the impact of (1) large‐scale factors, such as the *Fagus* species and the two climate variables temperature and precipitation; (2) stand scale factors such a forest management; and (3) and small‐scale factors such as the type of deadwood, decay stage, and diameter of the tree objects. For this, we used the concept of Hill numbers and focused on rare, common and dominant species (Hill, 1973). The Hill number concept was applied for the tree‐object scale (α‐diversity) and the scale of overall production and old‐growth beech forests of both *Fagus* species (γ‐diversity).

## 2 MATERIALS AND METHODS

### 2.1 Study areas

We collected drilling samples from 215 deadwood objects in different type of deadwood in production and old‐growth beech dominated forest stands in six countries during the 2021–2022 summer and autumn seasons: France, Germany, Ukraine, Bulgaria, Georgia, and Armenia. Bulgaria is the only country with both species of *Fagus*, where European beech from west to east is replaced by Oriental beech. The 18 forests investigated ranged from 65 to 1770 m above sea level (Table 1). For each plot, we extracted the extracted and used the local climatic variables mean annual temperature BIO1 and annual precipitation (BIO12) from WorldClim (Hijmans et al., 2005) grid data in 30 s resolution and calculated the mean values for a 1‐km radius around sampled localities, following the methodology of Gossner et al. (2013). Both variables were correlated with a Pearson's correlation coefficient of −.54, allowing simulatanouse use in one model.

**TABLE 1:** Description of study sites and samples.

Country	Region	Region2	Forest type	Label	Tree species	Latitude	Longitude	Elevation	Samples	Diameter [cm]	Decay	Types
France	Montreich	FRA_Fs	Production	FrMPFs	Fs	42.98643	0.97321	811–984	11	30–45	2–4	D, S
France	Montreich	FRA_Fs	Old‐growth	FrMOFs	Fs	42.99055	0.96659	951–958	11	30–75	2–4	D, S
Germany	Mittesteighuette	GER_Fs	Old‐growth	GrMOFs	Fs	49.09767	13.24844	718–774	17	60–120	1–4	D, S
Germany	Scheuereck	GER_Fs	Production	GrSPFs	Fs	49.06692	13.30664	765–831	14	22–78	1–4	D, S
Germany	Steigerwald	GER_Fs	Production	GrTPFs	Fs	49.93008	10.55793	438–475	19	18–84	1–4	D, S, St
Germany	Steigerwald	GER_Fs	Old‐growth	GrTOFs	Fs	49.86061	10.49864	355–404	20	60–120	2–4	D, S
Bulgaria	Berkovitsa	BUL_Fs	Old‐growth	BuBOFs	Fs	43.15803	23.12848	600–1465	11	30–62	3–4	D
Bulgaria	Strandhza	BUL_Fo	Old‐growth	BuSOFs	Fo	42.10796	27.78437	65–471	18	16–50	2–4	D, S
Ukraine	Mala Uholka	UKR_Fs	Old‐growth	UkMOFs	Fs	48.26886	23.62126	742–799	22	18–72	1–4	D, S
Ukraine	Velyka Uholka	UKR_Fs	Production	UkVPFs	Fs	48.22146	23.65095	325–530	21	10–58	1–4	D, S
Georgia	Borjomi	GEO_Fo	Production	GeBPFo	Fo	41.96267	43.44907	880–1113	10	50–92	1–4	D, S
Georgia	Borjomi	GEO_Fo	Old‐growth	GeBOFo	Fo	41.97949	43.45001	994–1160	9	26–46	1–4	D, S
Georgia	Lagodekhi	GEO_Fo	Old‐growth	GeLOFo	Fo	41.84729	46.32148	480–1005	11	28–52	1–3	St
Georgia	Mtisdziri	GEO_Fo	Production	GeMPFo	Fo	41.91701	46.09561	770–1128	13	40–82	1–4	S
Armenia	Antaramej	ARM_Fo	Old‐growth	ArAOFo	Fo	40.66068	45.07848	1768–1770	2	30–44	3–4	S, St
Armenia	Teghut	ARM_Fo	Old‐growth	ArTOFo	Fo	41.09047	44.81056	1120–1042	2	51–75	1–3	D
Armenia	Yenokavan	ARM_Fo	Old‐growth	ArYOFo	Fo	40.90897	45.06504	1388	1	22–22	3–3	St
Armenia	Zikatar	ARM_Fo	Old‐growth	ArZOFo	Fo	41.12143	44.92292	1280	3	32–42	2–3	D, S

*Note*: FRA, France; GER, Germany; BUL, Bulgaria; UKR, Ukraine; GEO, Georgia; and ARM, Armenia; Fs, *Fagus sylvatica*; Fo, *Fagus orientalis*; Types: D, downed deadwood; S, standing deadwood; and St, stump.

### 2.2 Field sampling

During the field sampling, we selected 10 deadwood beech items per site. Each item was a natural snag, natural log, or simply a stump. We chose only beech deadwood to exclude tree species effects beyond the genus *Fagus*. For each deadwood item, we recorded the type of deadwood (snag/standing tree, log, and stump), the diameter at breast height (DBH), the decay class (1–4; early, late early, middle, and late) (Müller‐Using & Bartsch, 2009), the coordinates (WGS84 world), the elevation and the *Fagus* species.

### 2.3 Fungal sampling

For collection of the molecular fungal community, we followed Rieker et al.'s (2022) sample protocol using disposable laboratory gloves (fresh pair for each item and disinfection before sampling), a knife for removing the outer bark surface of drilling position (to avoid contamination by random attached fragments and spores), spray bottle with 75% ethanol to disinfect the gloves and the drill after each object, Bunsen burner for flaming the drill and knife, the cordless drill with auger bits (10 mm diameter, 300 or 400 mm lengths), object with a diameter above 38 cm and then finally we drilled from both sides. We used Ziploc bags for collecting and storing the samples and labeling and a cool box with cold packs for transportation. The box was immediately placed inside the freezer at −20°C.

### 2.4 Laboratory work

#### 2.4.1 DNA extraction and isolation

The total community DNA was extracted from 0.150 g homogenized wood samples using NucleoSpin Soil, Mini kit for DNA (MACHEREY‐NAGEL GmbH & Co. KG, Düren, Germany) following the manufacturer's protocol. Bead beating was run on a FastPrep‐24 instrument (MPBiomedicals; 2 cycles of 30 s at speed 6.5). DNA concentrations were quantified using a NanoDrop UV–Vis spectrophotometer (Peqlab Biotechnologie GmbH, Erlangen, Germany). For sequencing the internal transcribed spacer (ITS2) regions of the fungal 18S rRNA gene, we applied for two‐step, Nextera barcoded PCR libraries using the locus specific primer pair ITS3 (5′‐GCA TCG ATG AAG AAC GCA GC‐3′) and ITS4 (5′‐TCC TCC GCT TAT TGA TAT GC‐3′) with 20 PCR cycles for the first step and 15 PCR cycles for the second step were created. Subsequently the PCR libraries were sequenced on an Illumina MiSeq platform using a v2 500 cycles kit.

#### 2.4.2 Amplicon‐metagenomics data analysis

The produced paired end reads, which passed Illumina's chastity filter, were subject to de‐multiplexing and trimming of Illumina adaptor residuals using Illumina's bcl2fastq software version v2.20.0.422. The quality of the reads was checked with the software FastQC version 0.11.8 and sequencing reads that fell below an average *Q*‐score of 24 or had any uncalled bases (*N*) were removed from further analysis. The locus specific ITS2 primers were trimmed from the sequencing reads with the software cutadapt v3.2. Paired‐end reads were discarded if the primer could not be trimmed. Trimmed forward and reverse reads of each paired‐end read were merged to *in‐silico* reform the sequenced molecule considering a minimum overlap of 15 bases using the software USEARCH version 11.0.667. Merged reads that contained ambiguous bases or were outliers regarding the expected amplicon size distribution were also discarded. Samples that resulted in less than 5000 merged reads were also discarded, to not distort the statistical analysis. From the remaining reads the fungal ITS2 subregions were extracted with help of the ITSx software suite v1.1.3 and its included database. The surviving reads were denoised using the UNOISE algorithm implemented in USEARCH to form zero‐radius OTUs (zOTUs) also named amplicon sequence variants (ASVs) discarding singletons and chimeras in the process. The resulting OTU abundance table was then filtered for possible barcode bleed‐in contaminations using the UNCROSS algorithm. OTU sequences were compared to the reference sequences of the UNITE database provided by https://www.drive5.com/usearch/manual/sintax_downloads.html, taxonomies were predicted and confidences were calculated using the SINTAX algorithm implemented in USEARCH. The identification revealed very similar proportions of unidentified OTUs with 43% in *Fagus orientalis* and 40% in *Fagus sylvatica* (Table S1). DNA extraction, library construction, sequencing and data analysis described in this section were performed by Microsynth AG (Balgach, Switzerland). For a list of OUT identifications, see Table S1.

#### 2.4.3 Statistical analyses

All statistical analyses were performed using R 4.3.1 (R Core Team, 2021). To determine fungal species richness and community composition, we followed two approaches. First, we used the observed species after excluding the records with only one read (Adamo et al., 2020). Removal of these records increase data quality for further analysis (Tedersoo et al., 2022). Second, we rarefied each community matrix (function *rrarefy*, package *vegan* by Oksanen et al., 2020). To determine a suitable rarefaction depth, we first calculated the read sums for each sample. Based on this exercise, we decided to use a minimum of 990 reads per samle as rarefaction depth. As diversity analyses of communities from both approaches revealed very similar results, we present the results only for the observed reads (first approach). We then calculated species richness for each object. Here, we are aware that OTUs are not equivalent to species but for reasons of readability, we chose the term species throughout the manuscript. Community matrices based on each log was calculated along the Hill numbers in “ecodist” package (Goslee & Urban, 2007) for dissimilarity indices representing a focus on rare (*q* = 0, Jaccard Index), common (*q* = 1, Horn Index) and dominant (*q* = 2, Morisita Horn) species (Chao et al., 2014). This allowed giving increasing weights to species with high abundances.

Different predictor sets for fungi community compositions at the object level were tested using multiple regression on distance matrices (MRMs) (Lichstein, 2007) as follows: First distance matrices for the fungi composition for *q* = 0, 1, and 2 were created. In a second step, distance matrices were created for the management type (production/old‐growth forests, Gower's distance), diameter of the deadwood object (Euclidian distance), temperature (Euclidian distance), precipitation (Euclidian distance), decay stage (Euclidian distance) and *Fagus* species (Gower distance). Since the two species are distributed along a longitudinal gradient, the predictor distance beech species was replaced in a second approach by a spatial distance (Euclidian distance) that considers the nested structure of the plots in the stands (Mamadashvili et al., 2023).

To model the species richness per deadwood object, we used a multiple negative binomial model as species numbers are count data. We included sampling site as a random factor to account for replicated measurements of different objects in a forest. As predictors, we used *Fagus* species, temperature, precipitation, forest type, deadwood type, wood decay and diameter (see Table S1). We finally repeated all analyses based on the rarefied communities (see above). As this did not change the results, we present these results only in the supplement.

To compare the gamma diversity of wood‐inhabiting fungi in production and old‐growth forests of both *Fagus* species, we fitted rarefaction‐extrapolation curves across all objects of each category based on the incidences of fungi species per object using the function iNext in package iNext (Hsieh et al., 2016). To account for unequal sample coverage in the four categories due to variation in sampling size or even natural variation in sample completeness, we standardized by sample coverage as recommended by Chao and Jost (2012). Non‐overlapping confidence intervals indicate significant differences.

## 3 RESULTS

**FIGURE 1:**
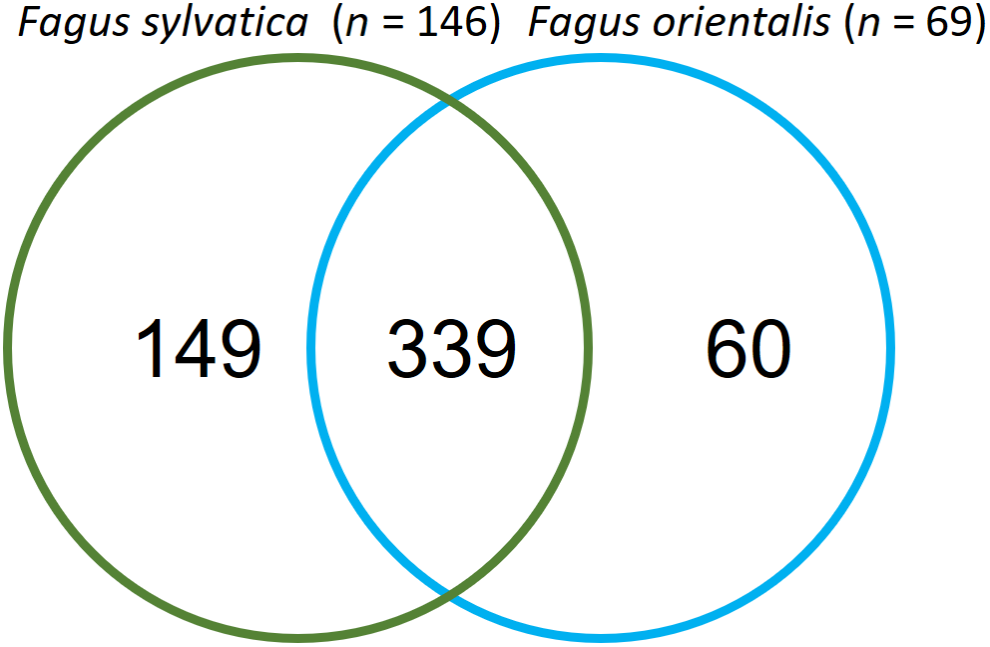
Venn diagram of fungal OTUs in the two *Fagus* species collected in 18 beech dominated forests, *n*, deadwood items sampled.

In total, we found 548 OTUs in 215 deadwood objects of beech. European beech revealed more unique OTUs than Oriental beech, but the majority (62%) could be found in both (Figure 1). Moreover, it is important to note that twice as many deadwood items were sampled in European beech (*n* = 146) than in Oriental beech (*n* = 69) sampling sites (Figure 1).

**FIGURE 2:**
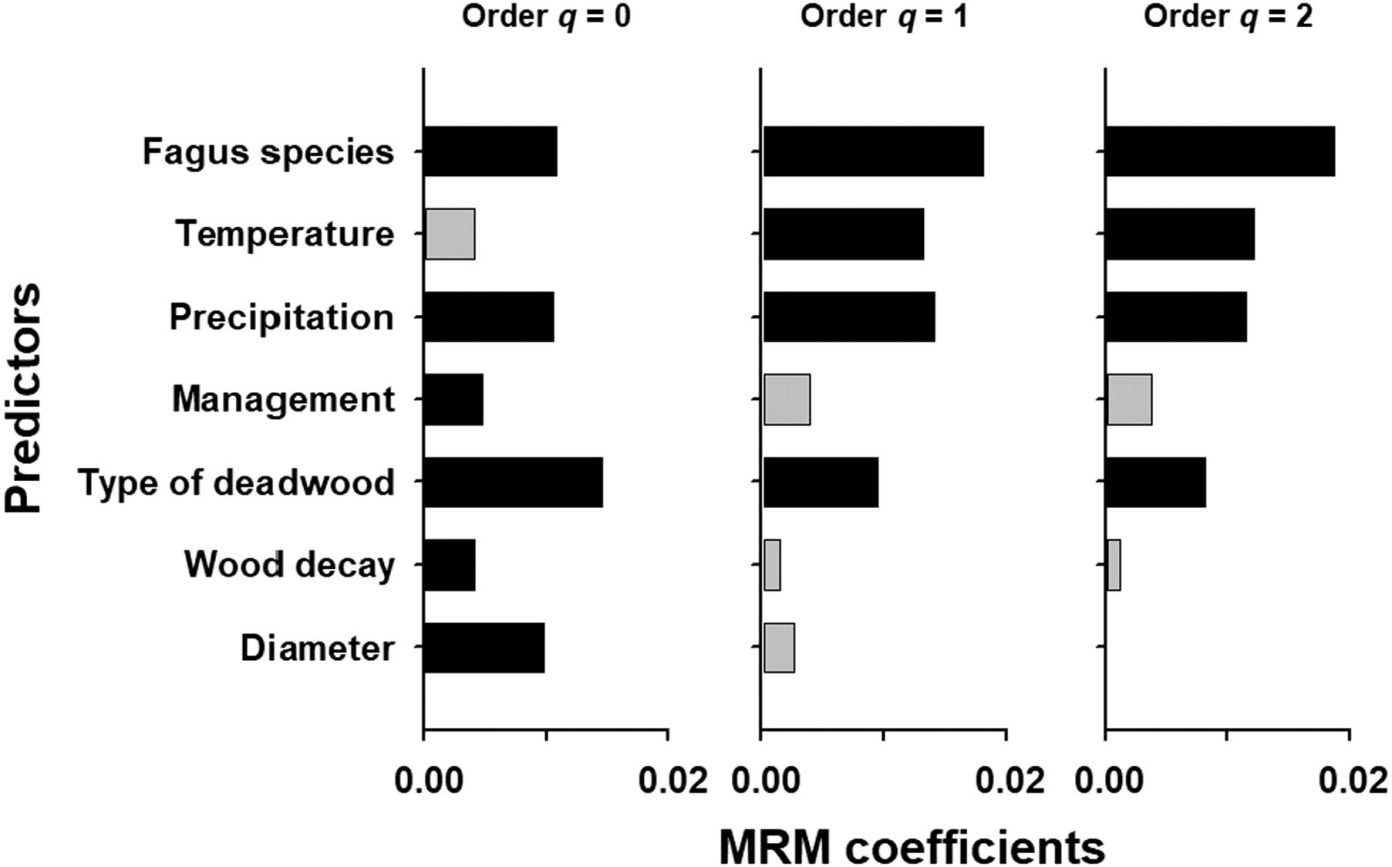
Multiple regression on distance matrices (MRM) coefficients of the predictors of the composition of fungi in 215 deadwood objects located in 18 beech dominated forests. Black bars indicate significance in MRM, with *p* < .05. Results are shown for rare (*q* = 0), common (*q* = 1) and dominant (*q* = 2) fungi species, gray‐nonsignificant. *Management* compared production with old‐growth forest stands, and *Fagus species* compared *F*. *sylvatica* with *F*. *orientalis*.

**FIGURE 3:**
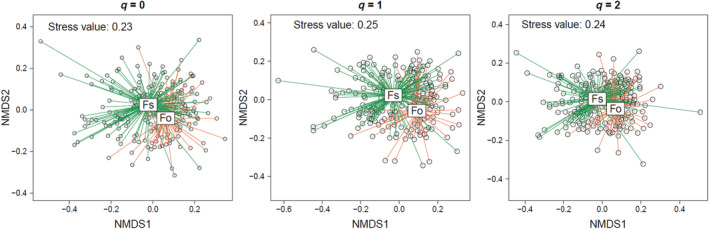
Community composition of fungi species identified by meta‐barcoding from 215 beech deadwood objects collected from *Fagus sylvatica* (Fs) and *Fagus orientalis* (Fo). Distance matrix for *q* = 0 is based on Jaccard Index, for *q* = 1 on Horn, and for *q* = 2 on Morisita‐Horn as in Figure 2.

Overall, the explained variance in fungal composition on the single object was low (0.22%–0.35%), however, the multiple regression on distance matrices, identified significant environmental variables determining the community composition (Figure 2). For q = 0, the *Fagus* species, the type of deadwood, the diameter, the precipitation, and management were identified as significant variables in descending order. With increasing Hill‐numbers, only *Fagus* species, temperature, precipitation, and type of deadwood remained significant determinants for community composition (Figure 2). However, as illustrated in Figure 3, the communities of both species showed a large overlap in wood‐inhabiting fungi. Substituting the distance matrix *Fagus* species by a spatial distance matrix revealed similar results (Figure S1), indicating that the effect of *Fagus* species and space cannot be distinguished due to the biogeographical distribution of the two *Fagus* species. These results were robust even when using the rarified species community (see R code in SM and Figure S2).

The richness of the single deadwood object (α‐diversity) was more or less independent from our predictors with one exception. With increasing decay stage, the richness per object increased significantly, which was again robust to the use of raw or rarified communities (Table S2).

**FIGURE 4:**
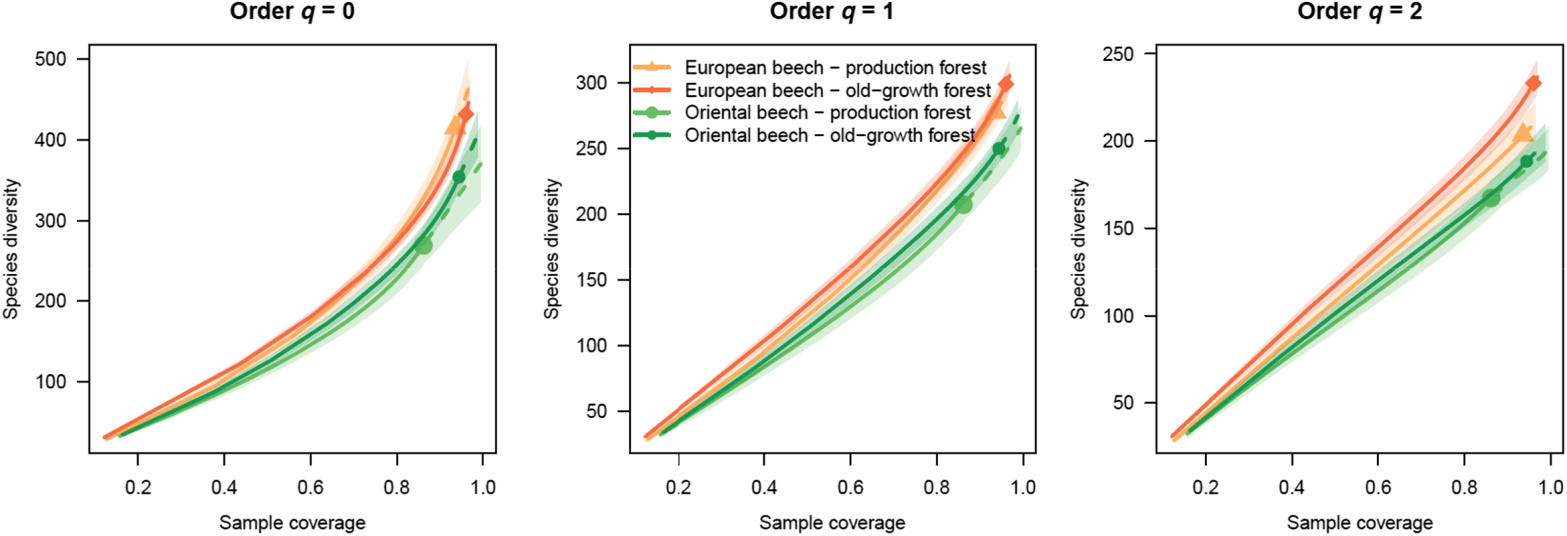
Gamma diversity of Beech forests of both *Fagus* species and in production as well as old‐growth sites using rarefaction‐extrapolation curves based on sample coverage. Solid lines indicate rarefaction, dashed lines extrapolation curves. Nonoverlapping confidence bands indicate significant difference.

Grouping all objects in four categories made by the combination of two *Fagus* species and the two management status (old‐growth vs. production), the rarefaction‐extrapolation curves showed higher γ‐diversity for both groups from European beech than in Oriental beech with focus on rare species at the same sample coverage (Figure 4, *q* = 0). Focusing on dominant species, the highest diversity was found in old‐growth European beech forests (Figure 4, *q* = 2).

## 4 DISCUSSION

Our systematic investigation of wood‐inhabiting fungi in beech forests from France to Armenia revealed overall high similarity in species composition and a difference in α‐diversity only by differences in decay stages. However, the species composition in European and Oriental beech revealed to be significantly different for all Hill numbers. Second, preciptation determined the species composition in all three Hill numbers. This underlines that large‐scale drivers including the both *Fagus* species and climate drive the overall very similar community of wood‐inhabiting fungi. Local factors as deadwood type, decay stage and diameter were only relevant for distinguishing composition of rare species.

### 4.1 Fagus species is important

Current research of fungal communities related to different host species compared mostly different host tree genera showing distinct fungal communities (Abrego, Norberg, et al., 2017; Englmeier et al., 2023; Krah et al., 2018; Purahong et al., 2018). Therefore, our knowledge on differences in fungi within a host genus is rather scarce. Here our findings are a first confirmation that fungal species might differ even between very similar tree species within a genus. However, if looking at the distribution of OTUs between the two Fagus species and taking into account the OTUs that could be detected in at least 10 samples, there were hardly any OTUs that only occur in one of the two Fagus species (Table S1). If this was the case, it was mostly unidentified species and exclusively on *Fagus sylvatica* but not on *Fagus orientalis*. This emphasizes that there are hardly any more frequently occurring unique fungal species/OTUs in *Fagus orientalis* to be expected.

The limitation for identifying the role of host tree identity versus biogeography remains in our study, because both beech species are spatially separated from West to East. Hence, finally we cannot distinguish between the impact of bioregion, history (Tertiary relicts in Oriental beech), and the host species and its traits itself. However, we can state that there are differences in fungi communities in deadwood of both species. Even earlier studies in European beech forests by Heilmann‐Clausen et al. (2014) demonstrated a longitudinal effect on the composition of fungi in European beech forests and was discussed as an effect of differences in climate and land use history. Finally it is important to consider that some studies have shown that climate influence wood properties in *F. orientalis* (Topaloğlu et al., 2016) or in both *Fagus* species (Elzami, 2018), which might affect the fungal composition.

We focused in this study on fungi species identified only via metabarcoding of wood samples. Studies on host effects with sporocarp surveys and metabarcoding revealed always a strong effect of the host identity on community composition in sporocarp datasets (Müller et al., 2020). Here sporocarp records represent probably more the dominant species in deadwood objects. This is further supported by the fact that the impact of host species for fungi by metabarcoding increased toward common and dominant species along the Hill numbers in Müller et al. (2020). Similarly, in our data the role of the *Fagus* species increased toward dominant species (Figure 2). In summary, we found either relatively similar communities or more diverse communities on the α‐ or on the γ‐level in much younger European beech forests than in the Tertiary relicts of Oriental beech forests.

### 4.2 Precipitation is always important, temperature toward dominant species

A second important variable for determining fungi community composition, for all Hill numbers, was precipitation (Figure 2). In contrast, the temperature proved to be important only for communities focusing on typical and dominant species (Figure 2). Beech forests form very different temperate forest ecosystems from lowland to montane levels in both European and Oriental beech. With increasing elevation, the precipitation regularly increases and temperature decreases which is one of the fundamental drivers for fungal composition (Bässler, Müller, Dziock, et al., 2010; Xu et al., 2023). Similarly, Heilmann‐Clausen et al. (2014) identified elevation as an important driver for turnover in fungal species communities within European beech reserves in Europe as well. In contrast, along a local elevation gradient Bässler, Müller, Dziock, et al. (2010) found structural parameters more important than the elevation gradient, which could not be confirmed in our large‐scale study. However, in most of these local studies (Bässler, Müller, Hothorn, et al., 2010) temperature and precipitation are to highly correlated to be distinguished. Here, our wide range of plots and only limited correlation of both, allowed to identify precipitation as more important in general and particularly for rare species communities, while the dominant communities were affected by both. This contrasts with studies from central Europe using wood‐inhabiting fungi in specific (Heilmann‐Clausen et al., 2014) and overall fungal diversity considering a broad range of guilds (Andrew et al., 2018). However, our spatial scale clearly exceeds the scale used in these studies which might explain the observed difference. Indeed, within increasing scale, both precipitation and temperature become important and have been suggested to drive global fungal diversity (Mikryukov et al., 2023; Tedersoo et al., 2014; Větrovský et al., 2019). As climate changes, temperature and precipitation are changing and many species shift their geographic range in these long‐lasting ecosystems (Antão et al., 2022). From our findings, this is to be expected also for wood‐inhabiting fungi (Bässler et al., 2016; Bässler, Müller, Hothorn, et al., 2010).

### 4.3 Gamma diversity of managed and unmanaged beech forests of both species

**FIGURE 5:**
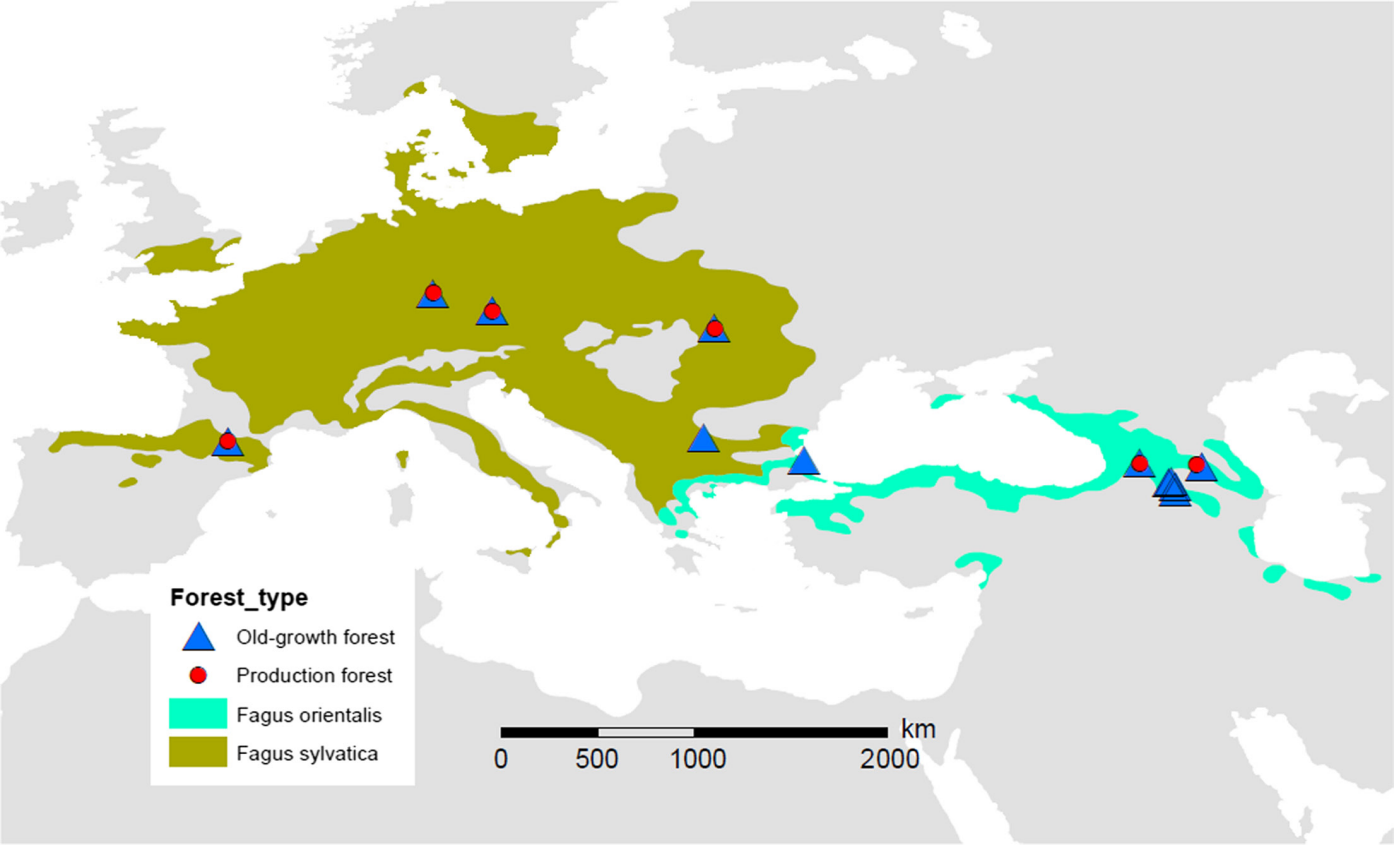
Geographical distribution of European (*Fagus sylvatica*) and Oriental (*F. orientalis*) beech (Caudullo et al., 2017) and the location of the 18 sampling stands (old‐growth and production forest stands) extending from the French Pyrenees to Armenia‐Caucasus, investigated for saproxylic fungi communities in deadwood.

Splitting the samples in both beech species and management types, we found the highest diversity of dominant species in old‐growth European beech forests, but not in production forests. No difference could be found between both management types in Oriental beech (Figure 4). This might indicate more impactful silviculture treatment in European beech than in Oriental beech forests, leading to reduction of functional diversity in the former in terms of deadwood (Gossner et al., 2013). However, the highest diversity in old‐growth European beech might be surprising as Oriental beech forests are in generally much older and some of them untouched at least since many centuries (Aliev, 2021; Kurz et al., 2023; UNESCO, 2013, 2019). Moreover, Oriental beech seems to be the older beech species harboring high levels of genetic diversity and should promote the diversity of its inhabitants (Azaryan et al., 2022; Cardoni et al., 2022; Kurz et al., 2023; Müller et al., 2016). On the other hand, the geographical range of European beech is much larger (see Figure 5) and in general larger host ranges lead to higher diversity (Brändle & Brandl, 2001). Additionally, the larger the range in European beech the broader the climate niche can be assumed, which then should promote diversity of fungi (Elzami, 2018). Although, climate niche studies did not confirm a broader niche width in Oriental versus European beech (Mellert & Šeho, 2022). Another reason might be larger phylogenetic tree diversity in European beech forests with oak in the lowlands and conifers as silver fir or spruce in higher altitudes, supporting more fungal species which might then jump over to beech deadwood (Krah et al., 2018). In contrast, most *Fagus orientalis* forests (with exception of Borjomi) lack conifers exhibiting in total a lower phylogenetic gamma diversity. On the other hand, wood‐inhabiting fungi are highly mobile. Therefore, historically younger old‐growth forests might be colonized successful by more dominant fungi species, which still coexist. In contrast, it might be that over the time since the Tertiary diversity of dominant fungi has shrunk as result of the high and long‐lasting competition. However, this suggestion remains to be confirmed in future studies, e.g. by experiments as in Englmeier et al. (2023).

Management affected the species composition of rare species, but not of common or dominant species. Here we have to keep in mind that this effect was controlled for structural elements as deadwood type, diameter, or decay stage, which are respectively influenced by forest management as well. This means that silvicultural management has comprehensive impacts on fungal composition at least for rare species. Such subtle effects of management on fungal communities have been shown along regional logging intensity gradients in European beech forests in Germany (Bässler et al., 2014; Müller, Engel, et al., 2007) or in Spain (Abrego & Salcedo, 2011), where fungal communities in old reserves differed than communities in production forests. These studies identified species promoted or negatively affected by increasing forest management (Abrego, Norberg, et al., 2017; Bässler et al., 2014).

### 4.4 Type of deadwood is important for the rare species

Different types of deadwood offer very different substrates, e.g., from wet to dry (microclimate) conditions. This opens different environmental conditions for different species. Therefore, forests with high variation of deadwood types provide more different niches for more species (Uhl et al., 2022). In contrast, even large amount of deadwood dominated by similar types as after large scale disturbance, can lead to reduced fungal diversity (Beudert et al., 2015), because wood‐inhabiting fungi are highly competitive (Boddy, 2021; Fukami et al., 2010). Regularly some dominant species occupy the major deadwood resources successfully and outcompete other species (Vogel et al., 2017). This has been shown on the scale of petri dishes in the lab (Fukami et al., 2010), to field experiments (Hagge et al., 2019) and indicated by global experiments (Seibold et al., 2021). As a consequence, fungi often show overdispersion in assembly patterns and have more species on more different objects as small twigs, when total resource volume is standardized (Bässler et al., 2014; Heilmann‐clausen & Christensen, 2004) and the decay is faster with a few dominant but efficient species than with a high diversity of species including many rare ones (Fukami et al., 2010). To escape the competition pressure, it seems therefore of advantage that deadwood is offered in many different types, which might have affected the composition in our data with focus on rare ones.

### 4.5 Diameter is important for the rare species

Bader et al. (1995) studied the deadwood size as an indicator for fungal diversity and showed that some species prefer well decayed and large logs, which is the reason why such species were well abundant in old growth forest sites but they became rare with increasing human activities‐cuttings. In addition, according to Küffer and Senn‐Irlet (2005) more species of fungi tend to be on deadwood with various diameters. However, Heilmann‐clausen and Christensen (2004) confirmed that the dead small trees and branches host higher diversity then large trees and larger logs do. Also, in production forest stands the main driver of deadwood fungi (and other saproxylic organisms) diversity is fine woody debris, which creates a general deadwood volume (Brabcová et al., 2022).

### 4.6 Wood decay stage as driver for species richness

Deadwood decomposition is a succession with distinct species turnovers over time in all organisms, including fungi (Fukasawa, 2018). Lindblad (1998), Fukasawa et al. (2009) and Pouska et al. (2016) found a significant effect of deadwood decay stage on fungi species richness and community composition. In systematic surveys by fruiting bodies and metabarcoding the decay stage was critical for fungal species composition along the Hill numbers (Müller et al., 2020). This is in contrast with our study, as we could not confirmed these findings with our results. Here the main reason might be that our sampling did not cover a broader range of decay or the pattern on the large scale was overridden by other factors. However, we found decay stage as the only predictor to increase α‐diversity per log (Table S1), which is in line with the findings from Bader et al. (1995) and Kubartová et al. (2012).

## 5 CONCLUSION FOR BIODIVERSITY CONSERVATION AND FOREST MANAGEMENT UNDER CLIMATE CHANGE

Our study provides some important implications for conservation and forest management. First, the high diversity and similarity of wood‐inhabiting fungi in beech forests across the 4k kilometer of temperate forest belt supports the view of a highly mobile organism group with a lot of functional insurance in rare and dominant species. From a mycological perspective the skepticism againts the usage of Oriental beech in silviculture managment in Western Europe seems not to be justified. Reason for that is the fact that fungi communities of both *Fagus* species are very similar. For conservation, our results show that effort should be put on establishment of protected areas in different climate conditions for both *Fagus* species, as currently mirrored in the Natural Heritages. For local managers interested in enhancing diversity of wood‐inhabiting fungi the retention of different types of beech deadwood seems promising.

## AUTHOR CONTRIBUTIONS

**Giorgi Mamadashvili:** Conceptualization (equal); data curation (lead); formal analysis (equal); writing – original draft (equal). **Antoine Brin:** Data curation (equal); resources (equal); writing – review and editing (equal). **Maksym Chumak:** Data curation (equal); resources (equal); writing – review and editing (equal). **Valeriia Diedus:** Data curation (equal); resources (equal); writing – review and editing (equal). **Lars Drössler:** Data curation (equal); resources (equal); writing – review and editing (equal). **Bernhard Förster:** Conceptualization (equal). **Kostadin B. Georgiev:** Data curation (equal); resources (equal); writing – review and editing (equal). **Tigran Ghrejyan:** Data curation (equal); resources (equal); writing – review and editing (equal). **Ruslan Hleb:** Data curation (equal); resources (equal); writing – review and editing (equal). **Mark Kalashian:** Data curation (equal); resources (equal); writing – review and editing (equal). **Ivan Kamburov:** Data curation (equal); resources (equal); writing – review and editing (equal). **Gayane Karagyan:** Data curation (equal); resources (equal); writing – review and editing (equal). **Joni Kevlishvili:** Data curation (equal); resources (equal); writing – review and editing (equal). **Zviad Khutsishvili:** Data curation (equal); resources (equal); writing – review and editing (equal). **Laurent Larrieu:** Data curation (equal); resources (equal); writing – review and editing (equal). **Meri Mazmanyan:** Data curation (equal); resources (equal); writing – review and editing (equal). **Peter I. Petrov:** Data curation (equal); resources (equal); writing – review and editing (equal). **Levan Tabunidze:** Data curation (equal); resources (equal); writing – review and editing (equal). **Claus Bässler:** Data curation (equal); resources (equal); writing – review and editing (equal). **Jörg Müller:** Data curation (equal); resources (equal); writing – review and editing (equal).

## CONFLICT OF INTEREST STATEMENT

The authors have no conflicts of interest.

## Supporting information


Data S1.

## Data Availability

Annotated R code, including the data needed to reproduce the statistical analyses and figures, is publicly available from figshare (DOI: 10.6084/m9.figshare.24720003). During review, code and data are available at: https://figshare.com/s/5e64d74d00e18a27f402.
