# Supplementary material for: Drivers of wood‐inhabiting fungal diversity in European and Oriental beech forests
Source: Ecol Evol. 2024 Jul 3;14(7):e11660. doi: 10.1002/ece3.11660 (PMC11220834; doi:10.1002/ece3.11660)
Supplement: Supplementary file 1 — Data S1. [file ECE3-14-e11660-s001.docx]

**Supplementary Material**

**Drivers of wood-inhabiting fungal diversity in European and Oriental beech forests**

Giorgi Mamadashvili, Antoine Brin, Maksym Chumak, Valeriia Diedus, Lars Drössler, Bernhard Förster, Kostadin B. Georgiev, Tigran Ghrejyan, Ruslan Hleb, Mark Kalashian, Ivan Kamburov, Gayane Karagyan, Joni Kevlishvili, Zviad Khutsishvili, Laurent Larrieu, Meri Mazmanyan, Peter I. Petrov, Levan Tabunidze, Claus Bässler, Jörg Müller

Tab. S1: Identification of OTUs at different levels and the respective samples in both Fagus species.

| OTU | Phylum | Order | Family | Spezies | sylvatica | orientalis |
| --- | --- | --- | --- | --- | --- | --- |
| OTU0001 | Basidiomycota | Polyporales | Polyporaceae | Fomes_fomentarius | 33 | 1 |
| OTU0002 | Ascomycota | unidentified | unidentified | Ascomycota_sp | 38 | 5 |
| OTU0003 | Ascomycota | unidentified | unidentified | Ascomycota_sp | 40 | 10 |
| OTU0004 | Ascomycota | Saccharomycetales | Debaryomycetaceae | Scheffersomyces_virginianus | 26 | 12 |
| OTU0005 | Basidiomycota | unidentified | unidentified | Agaricomycetes_sp | 19 | 2 |
| OTU0006 | Basidiomycota | Polyporales | Polyporaceae | Fomes_inzengae | 5 | 7 |
| OTU0007 | Basidiomycota | Polyporales | Polyporaceae | Fomes_fomentarius | 12 |  |
| OTU0008 | Ascomycota | Eurotiales | Aspergillaceae | Penicillium_sp | 13 | 10 |
| OTU0009 | Ascomycota | Helotiales | Mollisiaceae | Phialocephala_catenospora | 48 | 25 |
| OTU0010 | Ascomycota | Helotiales | Helotiaceae | Ascocoryne_albida | 28 | 5 |
| OTU0011 | Basidiomycota | Polyporales | Phanerochaetaceae | Bjerkandera_adusta | 4 | 5 |
| OTU0012 | Basidiomycota | unidentified | unidentified | Agaricomycetes_sp | 11 | 3 |
| OTU0013 | Ascomycota | Saccharomycetales | Trichomonascaceae | Sugiyamaella_sp | 13 | 7 |
| OTU0014 | Basidiomycota | Polyporales | Steccherinaceae | Ceriporiopsis_gilvescens | 17 | 6 |
| OTU0015 | Basidiomycota | Hymenochaetales | Rickenellaceae | Peniophorella_pubera | 13 | 4 |
| OTU0016 | Basidiomycota | Polyporales | Polyporaceae | Trametes_versicolor | 5 | 6 |
| OTU0017 | Ascomycota | Eurotiales | Aspergillaceae | Penicillium_sp | 23 | 13 |
| OTU0018 | Ascomycota | Hypocreales | Hypocreaceae | Trichoderma_sp | 30 | 7 |
| OTU0019 | Ascomycota | Capnodiales | Cladosporiaceae | Cladosporium_basi-inflatum | 21 | 19 |
| OTU0020 | Ascomycota | unidentified | unidentified | Ascomycota_sp | 25 | 5 |
| OTU0021 | Basidiomycota | Russulales | Stereaceae | Stereum_ostrea | 2 | 3 |
| OTU0022 | Ascomycota | Xylariales | Diatrypaceae | Eutypa_sp | 9 | 2 |
| OTU0023 | Ascomycota | Sordariales | Cephalothecaceae | Cryptendoxyla_hypophloia |  | 9 |
| OTU0024 | Basidiomycota | Trechisporales | Trechisporales_fam_Incertae_sedis | Sistotremastrum_guttuliferum | 5 | 3 |
| OTU0025 | Ascomycota | Xylariales | Xylariaceae | Xylaria_sp | 11 | 2 |
| OTU0026 | Basidiomycota | Cantharellales | Hydnaceae | Sistotrema_brinkmannii | 27 | 8 |
| OTU0027 | Basidiomycota | Polyporales | Polyporaceae | Trametes_versicolor | 8 | 3 |
| OTU0028 | Basidiomycota | Russulales | Stereaceae | Stereum_hirsutum | 6 | 6 |
| OTU0029 | Ascomycota | Saccharomycetales | Saccharomycetales_fam_Incertae_sedis | Candida_boleticola | 9 | 4 |
| OTU0030 | Basidiomycota | Polyporales | Ganodermataceae | Ganoderma_applanatum | 8 | 1 |
| OTU0031 | Basidiomycota | Cantharellales | Hydnaceae | Sistotrema_sp | 3 | 4 |
| OTU0032 | Basidiomycota | unidentified | unidentified | Basidiomycota_sp | 9 |  |
| OTU0033 | Basidiomycota | Polyporales | Podoscyphaceae | Hypochnicium_sp | 5 | 4 |
| OTU0034 | Basidiomycota | Polyporales | Phanerochaetaceae | Bjerkandera_adusta | 8 | 5 |
| OTU0035 | Ascomycota | Saccharomycetales | Trichomonascaceae | Sugiyamaella_sp | 18 |  |
| OTU0036 | Ascomycota | Sordariales | Cephalothecaceae | Cryptendoxyla_hypophloia | 28 | 2 |
| OTU0037 | Basidiomycota | Agaricales | Mycenaceae | Mycena_renati | 6 | 3 |
| OTU0038 | Basidiomycota | Polyporales | unidentified | Polyporales_sp | 8 | 3 |
| OTU0040 | Ascomycota | unidentified | unidentified | Ascomycota_sp | 35 |  |
| OTU0041 | Basidiomycota | Polyporales | Fomitopsidaceae | Fomitopsis_pinicola | 5 |  |
| OTU0043 | Basidiomycota | Thelephorales | Thelephoraceae | Amaurodon_viridis |  | 3 |
| OTU0044 | Basidiomycota | Polyporales | Polyporaceae | Trametes_versicolor | 1 | 1 |
| OTU0045 | Basidiomycota | Polyporales | Polyporaceae | Fomes_fomentarius | 1 |  |
| OTU0046 | Basidiomycota | Hymenochaetales | Schizoporaceae | Xylodon_flaviporus | 2 | 5 |
| OTU0047 | Basidiomycota | Hymenochaetales | Schizoporaceae | Xylodon_asperus | 1 |  |
| OTU0048 | Basidiomycota | Polyporales | Polyporaceae | Picipes_badius | 1 |  |
| OTU0050 | Basidiomycota | Hymenochaetales | Rickenellaceae | Peniophorella_pallida | 1 | 1 |
| OTU0051 | Basidiomycota | unidentified | unidentified | Agaricomycetes_sp |  | 2 |
| OTU0052 | Ascomycota | unidentified | unidentified | Ascomycota_sp | 19 |  |
| OTU0053 | Basidiomycota | unidentified | unidentified | Basidiomycota_sp | 13 | 11 |
| OTU0054 | Basidiomycota | unidentified | unidentified | Agaricomycetes_sp | 6 | 8 |
| OTU0055 | Ascomycota | Chaetosphaeriales | Chaetosphaeriaceae | Chaetosphaeriaceae_sp | 30 | 12 |
| OTU0057 | Ascomycota | Saccharomycetales | unidentified | Saccharomycetales_sp | 23 | 11 |
| OTU0058 | Basidiomycota | Agaricales | Cystostereaceae | Crustomyces_subabruptus | 7 | 1 |
| OTU0059 | Ascomycota | unidentified | unidentified | Ascomycota_sp | 53 | 23 |
| OTU0060 | Basidiomycota | Microbotryomycetes_ord_Incertae_sedis | Chrysozymaceae | Hamamotoa_singularis | 24 | 5 |
| OTU0061 | Ascomycota | Helotiales | Myxotrichaceae | Oidiodendron_sp | 7 | 5 |
| OTU0062 | Basidiomycota | Agaricales | Pleurotaceae | Pleurotus_ostreatus | 3 | 1 |
| OTU0063 | Ascomycota | Helotiales | unidentified | Helotiales_sp | 1 | 1 |
| OTU0064 | Ascomycota | Helotiales | Myxotrichaceae | Oidiodendron_periconioides | 12 | 13 |
| OTU0066 | Ascomycota | Saccharomycetales | Debaryomycetaceae | Scheffersomyces_ergatensis | 14 |  |
| OTU0067 | Ascomycota | Hypocreales | Hypocreaceae | Trichoderma_atroviride | 26 | 7 |
| OTU0068 | Ascomycota | Helotiales | Dermateaceae | Pezicula_eucrita |  | 1 |
| OTU0069 | Ascomycota | Capnodiales | Cladosporiaceae | Cladosporium_herbarum | 17 | 18 |
| OTU0070 | Basidiomycota | unidentified | unidentified | Agaricomycetes_sp |  | 2 |
| OTU0072 | Ascomycota | Dothideales | Saccotheciaceae | Aureobasidium_pullulans | 9 | 17 |
| OTU0073 | Basidiomycota | Hymenochaetales | Rickenellaceae | Peniophorella_pubera | 10 | 5 |
| OTU0075 | Basidiomycota | Russulales | Stereaceae | Stereum_ostrea | 3 | 1 |
| OTU0076 | Basidiomycota | Hymenochaetales | Schizoporaceae | Xylodon_chinensis | 7 |  |
| OTU0077 | Basidiomycota | Hymenochaetales | Rickenellaceae | Peniophorella_praetermissa | 7 | 3 |
| OTU0078 | Ascomycota | Eurotiales | Aspergillaceae | Penicillium_sp | 17 | 9 |
| OTU0079 | Ascomycota | Eurotiales | Aspergillaceae | Penicillium_sp | 21 | 4 |
| OTU0080 | Ascomycota | Helotiales | Helotiaceae | Scytalidium_lignicola | 15 | 20 |
| OTU0081 | Ascomycota | Saccharomycetales | Trichomonascaceae | Diddensiella_sp | 17 | 7 |
| OTU0082 | Basidiomycota | Agaricales | Mycenaceae | Mycena_crocata | 4 | 4 |
| OTU0083 | Basidiomycota | unidentified | unidentified | Basidiomycota_sp | 7 |  |
| OTU0084 | Ascomycota | Pleosporales | Cucurbitariaceae | Neocucurbitaria_irregularis | 4 | 19 |
| OTU0085 | Ascomycota | Xylariales | Hypoxylaceae | Hypoxylon_macrocarpum | 14 | 1 |
| OTU0086 | Ascomycota | Eurotiales | Aspergillaceae | Penicillium_sp | 34 | 11 |
| OTU0087 | Basidiomycota | Agaricales | Strophariaceae | Hypholoma_fasciculare | 6 | 1 |
| OTU0088 | Basidiomycota | Cystofilobasidiales | Mrakiaceae | Tausonia_pullulans | 24 | 9 |
| OTU0089 | Basidiomycota | Auriculariales | unidentified | Auriculariales_sp | 3 | 1 |
| OTU0090 | Ascomycota | Xylariales | Xylariaceae | Nemania_fusoidispora | 13 | 4 |
| OTU0091 | Basidiomycota | Hymenochaetales | Schizoporaceae | Schizopora_ovispora | 1 |  |
| OTU0092 | Ascomycota | Xylariales | Xylariaceae | Annulohypoxylon_sp | 14 |  |
| OTU0093 | Basidiomycota | unidentified | unidentified | Agaricomycetes_sp |  | 2 |
| OTU0094 | Basidiomycota | Agaricales | Mycenaceae | Mycena_galericulata | 2 | 1 |
| OTU0095 | Basidiomycota | Polyporales | Meruliaceae | Mycoacia_nothofagi | 10 | 2 |
| OTU0096 | Ascomycota | Saccharomycetales | Metschnikowiaceae | Metschnikowia_sp | 22 | 19 |
| OTU0098 | Basidiomycota | Polyporales | Meripilaceae | Rigidoporus_pouzarii |  | 1 |
| OTU0099 | Basidiomycota | Polyporales | Polyporaceae | Trametes_versicolor | 8 | 4 |
| OTU0100 | Ascomycota | Hypocreales | Hypocreaceae | Trichoderma_minutisporum | 14 | 5 |
| OTU0101 | Ascomycota | Helotiales | Mollisiaceae | Phialocephala_melitaea | 21 | 5 |
| OTU0102 | Basidiomycota | Auriculariales | Aporpiaceae | Elmerina_caryae | 7 | 4 |
| OTU0104 | Basidiomycota | Polyporales | Steccherinaceae | Ceriporiopsis_gilvescens | 4 | 4 |
| OTU0106 | Ascomycota | Chaetosphaeriales | Chaetosphaeriaceae | Chaetosphaeria_vermicularioides | 14 | 7 |
| OTU0107 | Basidiomycota | Filobasidiales | Piskurozymaceae | Piskurozyma_capsuligena | 6 |  |
| OTU0108 | Basidiomycota | Agaricales | Pleurotaceae | Pleurotus_ostreatus | 3 | 1 |
| OTU0109 | Basidiomycota | Tremellales | Carcinomycetaceae | Carcinomycetaceae_sp | 41 | 24 |
| OTU0111 | Basidiomycota | Polyporales | Hyphodermataceae | Hyphoderma_mutatum | 3 |  |
| OTU0112 | Ascomycota | Xylariales | Xylariaceae | Xylaria_sp | 5 |  |
| OTU0113 | Basidiomycota | Agaricales | Tricholomataceae | Megacollybia_platyphylla | 4 | 1 |
| OTU0114 | Ascomycota | Saccharomycetales | Trichomonascaceae | Sugiyamaella_sp | 8 |  |
| OTU0115 | Basidiomycota | Russulales | Russulales_fam_Incertae_sedis | Baltazaria_galactina | 3 | 1 |
| OTU0116 | Basidiomycota | Polyporales | Meruliaceae | Mycoacia_nothofagi | 5 |  |
| OTU0118 | Ascomycota | Chaetosphaeriales | Chaetosphaeriaceae | Chloridium_sp | 2 | 6 |
| OTU0119 | Ascomycota | Saccharomycetales | unidentified | Saccharomycetales_sp | 10 | 1 |
| OTU0120 | Basidiomycota | Agaricales | Tricholomataceae | Panellus_stipticus | 4 |  |
| OTU0121 | Ascomycota | unidentified | unidentified | Ascomycota_sp | 40 | 14 |
| OTU0122 | Basidiomycota | Russulales | Gloeocystidiellaceae | Gloeocystidiellum_bisporum | 1 | 2 |
| OTU0123 | Basidiomycota | Hymenochaetales | Schizoporaceae | Xylodon_chinensis | 2 |  |
| OTU0125 | Basidiomycota | Russulales | Stereaceae | Stereum_ostrea |  | 1 |
| OTU0126 | Ascomycota | Helotiales | unidentified | Helotiales_sp | 3 |  |
| OTU0127 | Basidiomycota | Hymenochaetales | Hymenochaetales_fam_Incertae_sedis | Trichaptum_biforme | 4 | 2 |
| OTU0128 | Ascomycota | Pleosporales | Pleosporaceae | Alternaria_prunicola | 14 | 21 |
| OTU0129 | Ascomycota | Xylariales | Diatrypaceae | Eutypa_lata |  | 2 |
| OTU0130 | Ascomycota | unidentified | unidentified | Ascomycota_sp |  | 6 |
| OTU0132 | Basidiomycota | unidentified | unidentified | Agaricomycetes_sp | 2 |  |
| OTU0133 | Basidiomycota | Polyporales | unidentified | Polyporales_sp | 2 |  |
| OTU0134 | Ascomycota | Saccharomycetales | Saccharomycetales_fam_Incertae_sedis | Kuraishia_floccosa | 9 | 1 |
| OTU0135 | Ascomycota | Eurotiales | Aspergillaceae | Penicillium_sp | 5 | 7 |
| OTU0136 | Ascomycota | Xylariales | Hypoxylaceae | Hypoxylon_papillatum |  | 1 |
| OTU0138 | Basidiomycota | Hymenochaetales | Schizoporaceae | Xylodon_raduloides | 3 | 1 |
| OTU0139 | Ascomycota | Chaetothyriales | Herpotrichiellaceae | Capronia_pilosella | 43 | 13 |
| OTU0142 | unidentified | unidentified | unidentified | Fungi_sp | 15 | 9 |
| OTU0143 | Basidiomycota | Trichosporonales | Trichosporonaceae | Apiotrichum_xylopini | 3 | 11 |
| OTU0144 | Basidiomycota | Polyporales | Polyporaceae | Perenniporia_narymica | 5 |  |
| OTU0145 | Basidiomycota | Cantharellales | Hydnaceae | Sistotrema_coroniferum | 2 |  |
| OTU0147 | Basidiomycota | Polyporales | Phanerochaetaceae | Bjerkandera_adusta | 4 |  |
| OTU0148 | Basidiomycota | Polyporales | Irpicaceae | Ceriporia_lacerata | 1 | 2 |
| OTU0149 | Basidiomycota | Agaricales | Tricholomataceae | Megacollybia_marginata | 1 |  |
| OTU0151 | Ascomycota | Eurotiales | Aspergillaceae | Penicillium_sp | 8 | 8 |
| OTU0152 | Ascomycota | unidentified | unidentified | Ascomycota_sp |  | 1 |
| OTU0153 | Ascomycota | Eurotiales | Aspergillaceae | Penicillium_sp | 3 | 2 |
| OTU0154 | Ascomycota | Sordariales | Helminthosphaeriaceae | Spadicoides_fuscolutea | 19 | 8 |
| OTU0155 | Ascomycota | Saccharomycetales | Alloascoideaceae | Alloascoidea_hylecoeti | 4 | 1 |
| OTU0156 | Ascomycota | Chaetothyriales | Herpotrichiellaceae | Cladophialophora_sp | 10 | 11 |
| OTU0157 | Ascomycota | Helotiales | Helotiaceae | Scytalidium_lignicola | 4 | 4 |
| OTU0158 | Ascomycota | unidentified | unidentified | Ascomycota_sp | 57 | 9 |
| OTU0159 | Basidiomycota | Polyporales | Phanerochaetaceae | Phanerochaete_sordida |  | 2 |
| OTU0160 | Basidiomycota | Polyporales | Phanerochaetaceae | Bjerkandera_adusta | 4 | 1 |
| OTU0161 | Ascomycota | Helotiales | Hyaloscyphaceae | Olla_transiens | 7 | 1 |
| OTU0162 | Basidiomycota | Hymenochaetales | Rickenellaceae | Peniophorella_praetermissa | 3 | 1 |
| OTU0163 | Basidiomycota | Auriculariales | Exidiaceae | Exidia_candida |  | 2 |
| OTU0164 | Ascomycota | Helotiales | unidentified | Helotiales_sp | 15 | 9 |
| OTU0165 | Basidiomycota | Russulales | Stereaceae | Stereum_ostrea | 3 |  |
| OTU0166 | Ascomycota | Sordariales | Helminthosphaeriaceae | Spadicoides_bina | 18 | 11 |
| OTU0167 | Ascomycota | Sordariales | unidentified | Sordariales_sp | 9 | 2 |
| OTU0168 | Ascomycota | Pleosporales | Melanommataceae | Melanomma_populicola | 8 | 4 |
| OTU0169 | Basidiomycota | Polyporales | unidentified | Polyporales_sp | 1 | 1 |
| OTU0171 | Basidiomycota | Auriculariales | Exidiaceae | Exidia_candida |  | 1 |
| OTU0172 | Mucoromycota | Mucorales | Cunninghamellaceae | Absidia_sp | 8 | 3 |
| OTU0173 | Basidiomycota | unidentified | unidentified | Agaricomycetes_sp | 3 |  |
| OTU0174 | Ascomycota | Eurotiales | Aspergillaceae | Aspergillaceae_sp | 24 | 10 |
| OTU0175 | Ascomycota | Chaetothyriales | Herpotrichiellaceae | Capronia_camelliae-yunnanensis | 42 | 21 |
| OTU0176 | Ascomycota | Eurotiales | Aspergillaceae | Penicillium_sp | 14 | 3 |
| OTU0177 | Ascomycota | Saccharomycetales | Trichomonascaceae | Sugiyamaella_sp | 1 | 4 |
| OTU0178 | Basidiomycota | Tremellales | Tremellaceae | Tremellaceae_sp | 10 | 5 |
| OTU0179 | Ascomycota | Saccharomycetales | Saccharomycetales_fam_Incertae_sedis | Candida_sp | 6 | 1 |
| OTU0180 | Basidiomycota | Russulales | Russulales_fam_Incertae_sedis | Baltazaria_galactina | 3 |  |
| OTU0181 | Basidiomycota | Polyporales | Hyphodermataceae | Hyphoderma_roseocremeum | 1 | 2 |
| OTU0183 | Basidiomycota | Hymenochaetales | Rickenellaceae | Peniophorella_praetermissa | 1 | 2 |
| OTU0184 | Basidiomycota | Hymenochaetales | Schizoporaceae | Xylodon_raduloides | 3 |  |
| OTU0186 | Ascomycota | Phaeomoniellales | Phaeomoniellaceae | Moristroma_germanicum | 1 | 1 |
| OTU0187 | Basidiomycota | Polyporales | Polyporaceae | Polyporus_tuberaster | 2 |  |
| OTU0188 | Basidiomycota | Trechisporales | Trechisporales_fam_Incertae_sedis | Trechisporales_fam_Incertae_sedis_sp | 3 |  |
| OTU0189 | Basidiomycota | Auriculariales | Exidiaceae | Exidia_glandulosa | 8 | 3 |
| OTU0190 | Ascomycota | Eurotiales | Aspergillaceae | Penicillium_sp | 26 | 4 |
| OTU0191 | Ascomycota | Mycosphaerellales | Teratosphaeriaceae | Teratosphaeriaceae_sp | 14 | 18 |
| OTU0192 | Basidiomycota | Agaricales | Mycenaceae | Mycena_semivestipes | 4 |  |
| OTU0193 | Basidiomycota | Hymenochaetales | Rickenellaceae | Peniophorella_guttulifera | 12 | 8 |
| OTU0194 | Ascomycota | Eurotiales | Aspergillaceae | Penicillium_sp | 10 | 11 |
| OTU0195 | Basidiomycota | Atractiellales | Phleogenaceae | Helicogloea_sebacea | 9 | 1 |
| OTU0196 | Basidiomycota | Agaricomycetes_ord_Incertae_sedis | Agaricomycetes_fam_Incertae_sedis | Xenasmatella_sp | 1 |  |
| OTU0198 | Basidiomycota | Agaricales | Cystostereaceae | Crustomyces_subabruptus | 5 | 1 |
| OTU0199 | Ascomycota | Capnodiales | Cladosporiaceae | Cladosporium_austrohemisphaericum | 17 | 21 |
| OTU0201 | Basidiomycota | Polyporales | Irpicaceae | Gloeoporus_dichrous |  | 1 |
| OTU0203 | Ascomycota | Saccharomycetales | Debaryomycetaceae | Scheffersomyces_illinoinensis | 5 | 8 |
| OTU0204 | Ascomycota | Hypocreales | unidentified | Hypocreales_sp | 12 | 5 |
| OTU0205 | Ascomycota | Saccharomycetales | Trichomonascaceae | Spencermartinsiella_europaea | 14 | 4 |
| OTU0206 | Ascomycota | Saccharomycetales | Metschnikowiaceae | Kodamaea_meredithae | 8 |  |
| OTU0207 | Basidiomycota | Auriculariales | Aporpiaceae | Elmerina_caryae | 2 |  |
| OTU0209 | Ascomycota | Saccharomycetales | Trichomonascaceae | Sugiyamaella_chiloensis | 15 | 6 |
| OTU0210 | Ascomycota | unidentified | unidentified | Lecanoromycetes_sp | 39 | 32 |
| OTU0212 | Ascomycota | Sordariales | Helminthosphaeriaceae | Spadicoides_hyalostoma | 14 | 4 |
| OTU0213 | Basidiomycota | Polyporales | Polyporaceae | Trametes_versicolor | 1 |  |
| OTU0214 | unidentified | unidentified | unidentified | Fungi_sp | 20 |  |
| OTU0215 | Basidiomycota | Hymenochaetales | Schizoporaceae | Xylodon_chinensis | 2 |  |
| OTU0216 | Basidiomycota | Polyporales | Irpicaceae | Gloeoporus_dichrous |  | 2 |
| OTU0217 | Ascomycota | Saccharomycetales | Trichomonascaceae | Blastobotrys_serpentis | 4 |  |
| OTU0218 | Basidiomycota | Auriculariales | Aporpiaceae | Elmerina_caryae | 1 |  |
| OTU0219 | Basidiomycota | Agaricales | Mycenaceae | Mycena_semivestipes | 7 |  |
| OTU0220 | Ascomycota | Xylariales | Graphostromataceae | Biscogniauxia_nummularia | 4 | 10 |
| OTU0221 | Ascomycota | Helotiales | Helotiaceae | Scytalidium_lignicola | 15 | 9 |
| OTU0222 | Ascomycota | Pleosporales | unidentified | Pleosporales_sp | 6 | 9 |
| OTU0223 | Ascomycota | Saccharomycetales | Debaryomycetaceae | Schwanniomyces_vanrijiae | 8 | 5 |
| OTU0224 | Basidiomycota | Polyporales | Meruliaceae | Scopuloides_hydnoides | 1 | 1 |
| OTU0225 | Ascomycota | unidentified | unidentified | Ascomycota_sp | 1 | 1 |
| OTU0226 | Ascomycota | Sordariales | Helminthosphaeriaceae | Spadicoides_bina | 17 | 9 |
| OTU0227 | Basidiomycota | Auriculariales | Aporpiaceae | Elmerina_caryae | 7 | 2 |
| OTU0229 | Ascomycota | Hypocreales | Niessliaceae | Niesslia_exilis | 1 |  |
| OTU0230 | Basidiomycota | unidentified | unidentified | Agaricomycetes_sp |  | 1 |
| OTU0231 | Basidiomycota | Agaricales | Crepidotaceae | Crepidotus_malachius | 4 | 4 |
| OTU0232 | Basidiomycota | Agaricales | Psathyrellaceae | Coprinellus_micaceus | 16 | 11 |
| OTU0233 | Ascomycota | Helotiales | Mollisiaceae | Phialocephala_collarifera | 8 | 1 |
| OTU0234 | Basidiomycota | Hymenochaetales | Schizoporaceae | Xylodon_sambuci | 3 |  |
| OTU0235 | Ascomycota | Chaetothyriales | Herpotrichiellaceae | Exophiala_sp | 18 | 12 |
| OTU0236 | Ascomycota | Sordariales | Cephalothecaceae | Cryptendoxyla_hypophloia | 10 | 7 |
| OTU0237 | Ascomycota | Hypocreales | Hypocreaceae | Trichoderma_bannaense | 1 | 1 |
| OTU0238 | Ascomycota | Chaetothyriales | Herpotrichiellaceae | Capronia_sp | 13 | 1 |
| OTU0239 | Ascomycota | Eurotiales | Aspergillaceae | Aspergillus_sp | 8 | 12 |
| OTU0240 | Ascomycota | Eurotiales | Aspergillaceae | Aspergillus_villosus | 13 | 5 |
| OTU0241 | Ascomycota | Helotiales | Sclerotiniaceae | Botrytis_caroliniana | 10 | 7 |
| OTU0242 | Basidiomycota | Hymenochaetales | Rickenellaceae | Peniophorella_pubera | 4 |  |
| OTU0243 | Ascomycota | Saccharomycetales | Saccharomycetales_fam_Incertae_sedis | Candida_trypodendri | 2 |  |
| OTU0244 | Basidiomycota | Amylocorticiales | Amylocorticiaceae | Plicaturopsis_crispa | 2 |  |
| OTU0245 | Basidiomycota | unidentified | unidentified | Basidiomycota_sp | 22 | 6 |
| OTU0246 | Ascomycota | Pleosporales | unidentified | Pleosporales_sp | 13 | 8 |
| OTU0247 | Ascomycota | Mycosphaerellales | Mycosphaerellaceae | Acrodontium_virellum | 5 |  |
| OTU0248 | Ascomycota | Ostropales | Stictidaceae | Cryptodiscus_sp | 21 | 11 |
| OTU0249 | Ascomycota | Pleosporales | Phaeosphaeriaceae | Jeremyomyces_labinae | 27 | 21 |
| OTU0250 | Ascomycota | Xylariales | Hypoxylaceae | Hypoxylon_invadens | 5 |  |
| OTU0251 | Ascomycota | Annulatascales | Annulatascaceae | Torrentispora_calembola | 17 | 8 |
| OTU0252 | Ascomycota | Pleosporales | Didymellaceae | Calophoma_rosae | 12 | 13 |
| OTU0253 | Ascomycota | Xylariales | Diatrypaceae | Diatrypaceae_sp |  | 3 |
| OTU0254 | Basidiomycota | Hymenochaetales | Schizoporaceae | Xylodon_chinensis | 6 |  |
| OTU0257 | Ascomycota | unidentified | unidentified | Sordariomycetes_sp | 3 | 1 |
| OTU0260 | Basidiomycota | Agaricales | Entolomataceae | Clitopilus_baronii | 7 | 7 |
| OTU0261 | Ascomycota | unidentified | unidentified | Ascomycota_sp | 40 | 38 |
| OTU0263 | Ascomycota | Pleosporales | Cucurbitariaceae | Neocucurbitaria_irregularis | 18 | 11 |
| OTU0265 | Ascomycota | Saccharomycetales | unidentified | Saccharomycetales_sp | 6 | 2 |
| OTU0266 | Ascomycota | Helotiales | Mollisiaceae | Mollisiaceae_sp | 7 | 1 |
| OTU0267 | Ascomycota | Hypocreales | Hypocreaceae | Trichoderma_deliquescens | 33 | 14 |
| OTU0268 | Basidiomycota | Agaricales | Strophariaceae | Pholiota_adiposa | 16 | 1 |
| OTU0269 | Basidiomycota | unidentified | unidentified | Agaricomycetes_sp |  | 1 |
| OTU0270 | Basidiomycota | Agaricales | Schizophyllaceae | Schizophyllum_commune | 2 | 2 |
| OTU0271 | Ascomycota | Eurotiales | Aspergillaceae | Aspergillus_sp | 12 |  |
| OTU0273 | Basidiomycota | Tremellales | Phaeotremellaceae | Phaeotremella_sp | 21 | 8 |
| OTU0274 | Ascomycota | Xylariales | Hypoxylaceae | Hypoxylon_rubiginosum | 4 | 5 |
| OTU0275 | Ascomycota | Chaetothyriales | Herpotrichiellaceae | Capronia_camelliae-yunnanensis | 40 | 24 |
| OTU0276 | Ascomycota | Ophiostomatales | Ophiostomataceae | Graphilbum_sp | 10 | 1 |
| OTU0277 | Ascomycota | Xylariales | Xylariaceae | Annulohypoxylon_sp | 7 |  |
| OTU0278 | Ascomycota | Helotiales | Hyaloscyphaceae | Olla_transiens | 25 | 3 |
| OTU0279 | Basidiomycota | Russulales | Hericiaceae | Dentipellis_fragilis | 3 |  |
| OTU0281 | Ascomycota | Pleosporales | Melanommataceae | Melanomma_pulvis-pyrius | 3 | 3 |
| OTU0282 | Ascomycota | Ostropales | Stictidaceae | Cryptodiscus_sp | 12 | 8 |
| OTU0283 | Basidiomycota | Sebacinales | unidentified | Sebacinales_sp |  | 4 |
| OTU0285 | Basidiomycota | Auriculariales | Hyaloriaceae | Stypella_subgelatinosa | 8 |  |
| OTU0286 | Ascomycota | Helotiales | Mollisiaceae | Phialocephala_sp | 6 | 2 |
| OTU0287 | Ascomycota | unidentified | unidentified | Ascomycota_sp | 18 | 6 |
| OTU0288 | Ascomycota | Hysteriales | Hysteriaceae | Hysterobrevium_constrictum |  | 5 |
| OTU0289 | Ascomycota | Eurotiales | Aspergillaceae | Penicillium_citreonigrum | 11 | 14 |
| OTU0290 | Basidiomycota | Cantharellales | unidentified | Cantharellales_sp | 13 | 2 |
| OTU0291 | Mortierellomycota | Mortierellales | Mortierellaceae | Mortierella_sp | 5 | 15 |
| OTU0293 | Basidiomycota | Auriculariales | Exidiaceae | Exidia_candida |  | 1 |
| OTU0294 | Basidiomycota | Agaricales | Mycenaceae | Mycena_semivestipes | 4 |  |
| OTU0295 | Ascomycota | Saccharomycetales | Debaryomycetaceae | Priceomyces_medius | 15 | 3 |
| OTU0296 | Ascomycota | Saccharomycetales | unidentified | Saccharomycetales_sp | 19 | 10 |
| OTU0297 | Ascomycota | Chaetothyriales | Herpotrichiellaceae | Capronia_sp | 24 | 15 |
| OTU0298 | Basidiomycota | Polyporales | Meruliaceae | Phlebia_tuberculata |  | 2 |
| OTU0299 | Ascomycota | Sordariales | unidentified | Sordariales_sp | 7 |  |
| OTU0300 | Ascomycota | Helotiales | Helotiaceae | Scytalidium_sp | 3 |  |
| OTU0301 | Basidiomycota | Auriculariales | Exidiaceae | Exidia_glandulosa | 7 |  |
| OTU0302 | Ascomycota | Saccharomycetales | Saccharomycetales_fam_Incertae_sedis | Suhomyces_anneliseae | 7 |  |
| OTU0303 | Mucoromycota | Mucorales | Cunninghamellaceae | Absidia_sp | 4 |  |
| OTU0304 | Basidiomycota | Polyporales | Meruliaceae | Phlebia_tuberculata | 5 | 1 |
| OTU0305 | Ascomycota | Eurotiales | Aspergillaceae | Penicillium_sp | 25 | 5 |
| OTU0306 | Mucoromycota | Mucorales | Mucoraceae | Mucor_abundans | 13 | 5 |
| OTU0307 | Basidiomycota | Polyporales | unidentified | Polyporales_sp | 1 |  |
| OTU0309 | Basidiomycota | Auriculariales | Aporpiaceae | Elmerina_caryae | 6 | 1 |
| OTU0310 | Basidiomycota | Corticiales | Corticiaceae | Corticiaceae_sp | 9 | 2 |
| OTU0311 | Basidiomycota | Polyporales | Hyphodermataceae | Hyphoderma_mutatum | 2 |  |
| OTU0312 | Ascomycota | Xylariales | Hypoxylaceae | Jackrogersella_cohaerens | 11 |  |
| OTU0313 | Ascomycota | Xylariales | Xylariaceae | Nemania_sp | 6 | 5 |
| OTU0315 | Ascomycota | unidentified | unidentified | Ascomycota_sp | 10 | 7 |
| OTU0316 | Ascomycota | Pleosporales | Nigrogranaceae | Nigrograna_antibiotica |  | 8 |
| OTU0317 | Mucoromycota | Umbelopsidales | Umbelopsidaceae | Umbelopsis_sp | 8 | 11 |
| OTU0318 | Basidiomycota | Atractiellales | Phleogenaceae | Helicogloea_compressa | 7 |  |
| OTU0319 | Ascomycota | Saccharomycetales | unidentified | Saccharomycetales_sp | 11 | 6 |
| OTU0322 | Ascomycota | Eurotiales | Aspergillaceae | Aspergillus_sp | 6 | 4 |
| OTU0323 | Basidiomycota | unidentified | unidentified | Agaricomycetes_sp | 1 | 1 |
| OTU0325 | Basidiomycota | Polyporales | Meruliaceae | Phlebia_tremellosa | 2 | 1 |
| OTU0326 | Ascomycota | unidentified | unidentified | Ascomycota_sp | 19 |  |
| OTU0327 | Ascomycota | unidentified | unidentified | Sordariomycetes_sp | 1 |  |
| OTU0328 | Ascomycota | Saccharomycetales | Trichomonascaceae | Sugiyamaella_sp | 9 |  |
| OTU0329 | Basidiomycota | Agaricales | Mycenaceae | Mycena_haematopus | 8 | 1 |
| OTU0331 | unidentified | unidentified | unidentified | Fungi_sp |  | 1 |
| OTU0332 | Ascomycota | Helotiales | Helotiaceae | Strossmayeria_bakeriana | 3 | 3 |
| OTU0333 | Ascomycota | unidentified | unidentified | Lecanoromycetes_sp | 26 | 22 |
| OTU0334 | Basidiomycota | Polyporales | Meruliaceae | Phlebia_radiata | 4 |  |
| OTU0335 | Basidiomycota | Sebacinales | unidentified | Sebacinales_sp |  | 2 |
| OTU0336 | Ascomycota | Eurotiales | Aspergillaceae | Penicillium_carneum | 3 | 1 |
| OTU0337 | Basidiomycota | Auriculariales | Auriculariales_fam_Incertae_sedis | Oliveonia_sp |  | 2 |
| OTU0338 | Ascomycota | Chaetothyriales | Herpotrichiellaceae | Cladophialophora_sp | 11 | 4 |
| OTU0340 | Basidiomycota | Hymenochaetales | Rickenellaceae | Peniophorella_praetermissa |  | 1 |
| OTU0341 | Basidiomycota | Atractiellales | Phleogenaceae | Helicogloea_aquilonia | 2 | 2 |
| OTU0342 | Ascomycota | Helotiales | Hyaloscyphaceae | Hyaloscypha_sp | 9 | 2 |
| OTU0343 | Ascomycota | Saccharomycetales | Debaryomycetaceae | Meyerozyma_amylolytica | 10 | 1 |
| OTU0345 | Ascomycota | Xylariales | Xylariaceae | Annulohypoxylon_sp | 7 |  |
| OTU0346 | Ascomycota | Chaetothyriales | Herpotrichiellaceae | Cladophialophora_sp | 5 | 18 |
| OTU0347 | Ascomycota | unidentified | unidentified | Ascomycota_sp | 21 | 1 |
| OTU0348 | Ascomycota | unidentified | unidentified | Ascomycota_sp | 5 | 6 |
| OTU0349 | Ascomycota | Phaeomoniellales | Phaeomoniellaceae | Moristroma_quercinum |  | 1 |
| OTU0350 | Ascomycota | Saccharomycetales | Pichiaceae | Martiniozyma_abietophila | 5 |  |
| OTU0351 | Basidiomycota | Polyporales | Meruliaceae | Phlebia_tuberculata | 2 | 2 |
| OTU0352 | Basidiomycota | unidentified | unidentified | Agaricomycetes_sp |  | 3 |
| OTU0353 | Basidiomycota | Auriculariales | Auriculariales_fam_Incertae_sedis | Oliveonia_sp |  | 1 |
| OTU0354 | Ascomycota | Pleosporales | unidentified | Pleosporales_sp | 12 | 13 |
| OTU0355 | Basidiomycota | Cystofilobasidiales | Mrakiaceae | Mrakia_blollopis | 3 | 6 |
| OTU0356 | Ascomycota | unidentified | unidentified | Ascomycota_sp | 11 |  |
| OTU0357 | Ascomycota | Helotiales | Dermateaceae | Pezicula_sporulosa | 5 |  |
| OTU0358 | Basidiomycota | Polyporales | Polyporaceae | Trametes_pubescens | 1 |  |
| OTU0359 | Ascomycota | Pezizales | Sarcoscyphaceae | Sarcoscypha_coccinea |  | 1 |
| OTU0360 | Ascomycota | Xylariales | Graphostromataceae | Biscogniauxia_nummularia | 4 | 2 |
| OTU0361 | Ascomycota | unidentified | unidentified | Dothideomycetes_sp |  | 1 |
| OTU0362 | Ascomycota | Saccharomycetales | Debaryomycetaceae | Yamadazyma_mexicana | 1 |  |
| OTU0364 | Ascomycota | Helotiales | Mollisiaceae | Phialocephala_collarifera | 7 | 1 |
| OTU0365 | Basidiomycota | Agaricales | Strophariaceae | Pholiota_adiposa | 1 |  |
| OTU0366 | Ascomycota | Ostropales | Ostropales_fam_Incertae_sedis | Mulderomyces_natalis | 9 | 8 |
| OTU0367 | Ascomycota | Chaetothyriales | Herpotrichiellaceae | Capronia_pilosella |  | 16 |
| OTU0368 | Basidiomycota | Polyporales | Meruliaceae | Phlebia_tremellosa | 1 |  |
| OTU0371 | Ascomycota | Eurotiales | Aspergillaceae | Penicillium_sp | 12 | 3 |
| OTU0372 | unidentified | unidentified | unidentified | Fungi_sp | 34 | 18 |
| OTU0374 | Ascomycota | Chaetothyriales | Herpotrichiellaceae | Veronaea_aquatica |  | 5 |
| OTU0375 | Basidiomycota | Sebacinales | Sebacinaceae | Chaetospermum_sp | 1 | 3 |
| OTU0376 | Ascomycota | Chaetothyriales | Herpotrichiellaceae | Rhinocladiella_quercus | 25 | 5 |
| OTU0378 | Ascomycota | Sordariales | unidentified | Sordariales_sp | 4 | 6 |
| OTU0379 | Ascomycota | Phaeomoniellales | Phaeomoniellaceae | Moristroma_palatinum | 1 |  |
| OTU0380 | Basidiomycota | Polyporales | Meruliaceae | Scopuloides_hydnoides | 8 | 1 |
| OTU0381 | Ascomycota | unidentified | unidentified | Ascomycota_sp | 11 | 5 |
| OTU0382 | Ascomycota | Pleosporales | Nigrogranaceae | Nigrograna_cangshanensis | 6 | 4 |
| OTU0383 | Ascomycota | Tubeufiales | Tubeufiaceae | Acanthostigma_chiangmaiense |  | 1 |
| OTU0384 | Ascomycota | Saccharomycetales | Trichomonascaceae | Blastobotrys_sp | 20 | 6 |
| OTU0385 | Ascomycota | Sordariomycetes_ord_Incertae_sedis | Sordariomycetes_fam_Incertae_sedis | Paradiplococcium_sp | 3 | 3 |
| OTU0386 | Basidiomycota | Amylocorticiales | Amylocorticiaceae | Plicaturopsis_crispa | 2 |  |
| OTU0387 | Ascomycota | Chaetothyriales | Herpotrichiellaceae | Capronia_sp | 6 | 12 |
| OTU0388 | Ascomycota | Xylariales | Xylariaceae | Annulohypoxylon_sp | 6 |  |
| OTU0389 | Basidiomycota | Cantharellales | Hydnaceae | Sistotrema_coronilla | 7 | 5 |
| OTU0391 | Basidiomycota | Phallales | Phallaceae | Phallus_impudicus | 2 | 13 |
| OTU0392 | Basidiomycota | Boletales | Coniophoraceae | Coniophora_puteana | 1 | 2 |
| OTU0394 | Ascomycota | unidentified | unidentified | Ascomycota_sp | 10 |  |
| OTU0395 | Ascomycota | Eurotiales | Aspergillaceae | Talaromyces_iowaense | 3 |  |
| OTU0396 | Ascomycota | Saccharomycetales | unidentified | Saccharomycetales_sp | 5 |  |
| OTU0397 | Basidiomycota | Agaricales | Mycenaceae | Mycena_metata | 2 |  |
| OTU0398 | Ascomycota | Sordariales | Helminthosphaeriaceae | Spadicoides_bina | 12 | 7 |
| OTU0399 | Ascomycota | Xylariales | Graphostromataceae | Biscogniauxia_nummularia | 2 |  |
| OTU0400 | Basidiomycota | Trechisporales | Trechisporales_fam_Incertae_sedis | Sistotremastrum_vigilans | 3 |  |
| OTU0401 | Mucoromycota | Umbelopsidales | Umbelopsidaceae | Umbelopsis_sp | 2 | 13 |
| OTU0402 | Basidiomycota | Agaricales | Mycenaceae | Mycena_haematopus | 1 |  |
| OTU0403 | Ascomycota | Chaetothyriales | Herpotrichiellaceae | Cladophialophora_sp | 33 | 13 |
| OTU0404 | Basidiomycota | Tremellales | Tremellales_fam_Incertae_sedis | Cuniculitrema_polymorpha | 12 | 5 |
| OTU0405 | Ascomycota | Sclerococcales | Sclerococcaceae | Rhopalophora_clavispora | 10 | 7 |
| OTU0406 | Ascomycota | Helotiales | Myxotrichaceae | Myxotrichum_sp | 8 |  |
| OTU0407 | Ascomycota | Ophiostomatales | Ophiostomataceae | Ophiostomataceae_sp | 5 |  |
| OTU0408 | Ascomycota | Pleosporales | Lophiotremataceae | Lophiotrema_sp | 2 |  |
| OTU0409 | Ascomycota | Eurotiales | Aspergillaceae | Paecilomyces_variotii | 1 |  |
| OTU0410 | Basidiomycota | Agaricales | Mycenaceae | Mycena_renati | 1 | 1 |
| OTU0411 | Ascomycota | Xylariales | Lopadostomataceae | Lopadostoma_fagi | 5 | 3 |
| OTU0412 | Mucoromycota | Umbelopsidales | Umbelopsidaceae | Umbelopsis_sp | 6 | 10 |
| OTU0413 | Ascomycota | Helotiales | Chlorociboriaceae | Chlorociboria_sp | 3 |  |
| OTU0414 | Basidiomycota | Polyporales | Meruliaceae | Phlebia_tuberculata |  | 1 |
| OTU0415 | Ascomycota | Chaetothyriales | Herpotrichiellaceae | Veronaea_aquatica | 3 | 8 |
| OTU0416 | Ascomycota | Saccharomycetales | Saccharomycetaceae | Ogataea_nitratoaversa | 1 | 2 |
| OTU0417 | Basidiomycota | Agaricales | Psathyrellaceae | Coprinellus_micaceus |  | 2 |
| OTU0419 | Ascomycota | Pleosporales | Cucurbitariaceae | Neocucurbitaria_quercina | 3 | 6 |
| OTU0420 | Ascomycota | Xenospadicoidales | Xenospadicoidaceae | Pseudodiplococcium_sp | 8 | 2 |
| OTU0421 | Ascomycota | Saccharomycetales | Trichomonascaceae | Blastobotrys_niveus | 19 | 9 |
| OTU0422 | Ascomycota | unidentified | unidentified | Lecanoromycetes_sp | 15 | 18 |
| OTU0423 | Basidiomycota | Microbotryomycetes_ord_Incertae_sedis | Chrysozymaceae | Hamamotoa_lignophila | 9 |  |
| OTU0424 | Basidiomycota | Cantharellales | Hydnaceae | Sistotrema_raduloides | 4 |  |
| OTU0425 | Basidiomycota | Tremellales | Syzygosporaceae | Syzygosporaceae_sp | 12 | 10 |
| OTU0426 | Basidiomycota | Polyporales | Polyporaceae | Daedaleopsis_confragosa | 1 |  |
| OTU0427 | Basidiomycota | Sporidiobolales | Sporidiobolaceae | Rhodosporidiobolus_colostri | 11 | 7 |
| OTU0428 | Basidiomycota | Polyporales | Podoscyphaceae | Hypochnicium_cremicolor | 1 | 2 |
| OTU0429 | Ascomycota | Pleosporales | Didymellaceae | Xenodidymella_camporesii |  | 1 |
| OTU0430 | Ascomycota | Botryosphaeriales | Botryosphaeriaceae | Lasiodiplodia_endophytica |  | 4 |
| OTU0431 | Basidiomycota | Polyporales | Meruliaceae | Scopuloides_sp | 1 |  |
| OTU0432 | Mucoromycota | Mucorales | Mucoraceae | Mucor_hiemalis | 8 | 8 |
| OTU0433 | Ascomycota | unidentified | unidentified | Sordariomycetes_sp |  | 8 |
| OTU0434 | Ascomycota | Helotiales | Myxotrichaceae | Oidiodendron_sp | 1 |  |
| OTU0435 | Basidiomycota | Auriculariales | Hyaloriaceae | Stypella_grilletii | 2 |  |
| OTU0437 | Ascomycota | Chaetothyriales | Herpotrichiellaceae | Exophiala_moniliae | 25 | 7 |
| OTU0438 | Ascomycota | Patellariales | Patellariaceae | Rhizodiscina_lignyota | 5 | 10 |
| OTU0439 | unidentified | unidentified | unidentified | Fungi_sp | 16 | 15 |
| OTU0440 | Ascomycota | Hypocreales | Hypocreaceae | Trichoderma_harzianum | 17 | 15 |
| OTU0441 | Ascomycota | Annulatascales | Annulatascaceae | Torrentispora_calembola | 3 |  |
| OTU0442 | Basidiomycota | Russulales | Auriscalpiaceae | Lentinellus_ursinus |  | 1 |
| OTU0443 | Basidiomycota | Hymenochaetales | Rickenellaceae | Peniophorella_pertenuis | 1 |  |
| OTU0444 | Ascomycota | unidentified | unidentified | Dothideomycetes_sp | 1 | 7 |
| OTU0445 | Ascomycota | Helotiales | Myxotrichaceae | Oidiodendron_sp | 6 | 1 |
| OTU0446 | Basidiomycota | Cantharellales | Hydnaceae | Sistotrema_raduloides | 1 |  |
| OTU0447 | Ascomycota | Pleosporales | unidentified | Pleosporales_sp | 1 |  |
| OTU0448 | Mucoromycota | Umbelopsidales | Umbelopsidaceae | Umbelopsis_sp | 9 | 6 |
| OTU0449 | Basidiomycota | Russulales | Gloeocystidiellaceae | Gloeocystidiellum_bisporum | 1 |  |
| OTU0450 | Basidiomycota | Agaricales | Mycenaceae | Mycena_metata | 1 |  |
| OTU0451 | Basidiomycota | Russulales | Stereaceae | Stereum_ostrea | 1 | 1 |
| OTU0453 | Basidiomycota | unidentified | unidentified | Agaricomycetes_sp | 5 | 23 |
| OTU0454 | Ascomycota | Pleosporales | Pleomonodictydaceae | Pleomonodictys_descalsii |  | 4 |
| OTU0455 | Ascomycota | Helotiales | Helotiaceae | Scytalidium_sp | 9 | 8 |
| OTU0456 | Basidiomycota | Auriculariales | Hyaloriaceae | Myxarium_hyalinum |  | 2 |
| OTU0457 | Basidiomycota | Polyporales | Phanerochaetaceae | Phanerochaete_sordida |  | 2 |
| OTU0458 | Ascomycota | Saccharomycetales | Saccharomycetales_fam_Incertae_sedis | Candida_chilensis | 1 |  |
| OTU0459 | Ascomycota | Chaetothyriales | Herpotrichiellaceae | Capronia_pulcherrima | 28 | 4 |
| OTU0460 | Ascomycota | Xylariales | unidentified | Xylariales_sp | 2 |  |
| OTU0461 | Ascomycota | Xylariales | Xylariaceae | Xylariaceae_sp | 3 |  |
| OTU0462 | Ascomycota | Dothideomycetes_ord_Incertae_sedis | Dothideomycetes_fam_Incertae_sedis | Catinella_olivacea | 13 | 6 |
| OTU0463 | Ascomycota | Xylariales | Xylariaceae | Kretzschmaria_deusta | 25 | 10 |
| OTU0465 | Ascomycota | Orbiliales | Orbiliaceae | Orbiliaceae_sp | 2 | 4 |
| OTU0466 | Ascomycota | Chaetothyriales | Herpotrichiellaceae | Exophiala_moniliae | 21 | 17 |
| OTU0467 | Ascomycota | Chaetosphaeriales | Chaetosphaeriaceae | Chloridium_sp | 22 | 16 |
| OTU0468 | Basidiomycota | Boletales | Coniophoraceae | Coniophora_puteana | 4 | 1 |
| OTU0469 | Basidiomycota | Amylocorticiales | Amylocorticiaceae | Plicaturopsis_crispa | 1 |  |
| OTU0470 | Basidiomycota | Atractiellales | Phleogenaceae | Helicogloea_sebacea | 1 |  |
| OTU0471 | Mucoromycota | Mucorales | Mucoraceae | Mucor_sp |  | 9 |
| OTU0472 | Ascomycota | unidentified | unidentified | Ascomycota_sp | 2 | 1 |
| OTU0473 | Basidiomycota | Atractiellales | Phleogenaceae | Helicogloea_pellucida | 14 | 6 |
| OTU0474 | Basidiomycota | Amylocorticiales | Amylocorticiaceae | Plicaturopsis_crispa | 1 |  |
| OTU0475 | Ascomycota | Pleosporales | Melanommataceae | Melanomma_populicola | 3 |  |
| OTU0476 | Ascomycota | Capnodiales | Capnodiales_fam_Incertae_sedis | Arthrocatena_sp | 3 | 5 |
| OTU0477 | Ascomycota | Helotiales | Amorphothecaceae | Amorphothecaceae_sp |  | 1 |
| OTU0478 | unidentified | unidentified | unidentified | Fungi_sp | 4 |  |
| OTU0480 | Ascomycota | unidentified | unidentified | Sordariomycetes_sp | 3 | 1 |
| OTU0481 | Ascomycota | Sordariales | Helminthosphaeriaceae | Spadicoides_bina | 5 | 3 |
| OTU0482 | Basidiomycota | Tremellales | Tremellales_fam_Incertae_sedis | Cuniculitrema_polymorpha | 4 | 3 |
| OTU0484 | Ascomycota | Phaeomoniellales | Phaeomoniellaceae | Moristroma_palatinum | 1 |  |
| OTU0485 | Basidiomycota | Agaricales | Strophariaceae | Pholiota_adiposa | 1 | 1 |
| OTU0486 | Ascomycota | Eurotiales | Aspergillaceae | Penicillium_virgatum | 21 | 9 |
| OTU0487 | Basidiomycota | Agaricales | Mycenaceae | Mycena_renati | 6 | 2 |
| OTU0488 | Ascomycota | Saccharomycetales | Debaryomycetaceae | Debaryomyces_sp | 7 | 2 |
| OTU0489 | Basidiomycota | Filobasidiales | Filobasidiaceae | Filobasidium_wieringae | 4 | 7 |
| OTU0490 | Basidiomycota | Agaricales | Strophariaceae | Pholiota_adiposa | 3 |  |
| OTU0491 | Ascomycota | Helotiales | Helotiaceae | Strossmayeria_bakeriana | 6 |  |
| OTU0493 | Ascomycota | Hysteriales | Hysteriaceae | Hysterobrevium_constrictum |  | 6 |
| OTU0494 | Basidiomycota | Hymenochaetales | Schizoporaceae | Schizopora_paradoxa | 1 |  |
| OTU0495 | Basidiomycota | Boletales | Coniophoraceae | Coniophora_puteana |  | 1 |
| OTU0496 | Ascomycota | unidentified | unidentified | Lecanoromycetes_sp | 2 |  |
| OTU0497 | Basidiomycota | Cantharellales | Hydnaceae | Sistotrema_sernanderi | 7 |  |
| OTU0498 | Ascomycota | Helotiales | unidentified | Helotiales_sp |  | 1 |
| OTU0499 | Ascomycota | Pleosporales | unidentified | Pleosporales_sp |  | 2 |
| OTU0500 | Ascomycota | unidentified | unidentified | Dothideomycetes_sp | 2 | 15 |
| OTU0501 | Basidiomycota | Exobasidiales | Brachybasidiaceae | Meira_sp | 4 | 3 |
| OTU0502 | Ascomycota | Pleosporales | unidentified | Pleosporales_sp | 8 | 3 |
| OTU0503 | Ascomycota | Pleosporales | unidentified | Pleosporales_sp | 2 | 1 |
| OTU0506 | Ascomycota | Sordariales | Helminthosphaeriaceae | Spadicoides_hyalostoma | 1 |  |
| OTU0507 | Ascomycota | Saccharomycetales | Trichomonascaceae | Blastobotrys_americanus | 7 |  |
| OTU0508 | Ascomycota | Xenospadicoidales | Xenospadicoidaceae | Calyptosphaeria_subdenudata |  | 2 |
| OTU0509 | Ascomycota | Sordariales | Cephalothecaceae | Phialemonium_sp | 1 |  |
| OTU0511 | Ascomycota | Eurotiales | Aspergillaceae | Talaromyces_sp | 1 |  |
| OTU0513 | Basidiomycota | Auriculariales | Exidiaceae | Exidia_truncata | 1 |  |
| OTU0514 | Basidiomycota | Cantharellales | Hydnaceae | Sistotrema_raduloides | 2 |  |
| OTU0515 | Basidiomycota | Agaricales | Cyphellaceae | Cyphellopsis_anomala | 5 |  |
| OTU0516 | Ascomycota | Helotiales | unidentified | Helotiales_sp | 4 | 1 |
| OTU0518 | Basidiomycota | Agaricales | Entolomataceae | Clitopilus_hobsonii | 3 |  |
| OTU0519 | Ascomycota | unidentified | unidentified | Ascomycota_sp | 15 |  |
| OTU0520 | Ascomycota | Pleosporales | Didymellaceae | Ascochyta_herbicola | 14 | 10 |
| OTU0521 | Ascomycota | Chaetothyriales | Herpotrichiellaceae | Capronia_sp | 14 | 5 |
| OTU0522 | Ascomycota | Sordariomycetes_ord_Incertae_sedis | Sordariomycetes_fam_Incertae_sedis | Lentomitella_cirrhosa | 9 | 3 |
| OTU0523 | Ascomycota | Orbiliales | Orbiliaceae | Orbilia_xanthostigma | 12 | 7 |
| OTU0524 | Ascomycota | Orbiliales | Orbiliaceae | Orbilia_sp | 1 | 3 |
| OTU0525 | Ascomycota | Hypocreales | Hypocreales_fam_Incertae_sedis | Barbatosphaeria_barbirostris | 16 | 4 |
| OTU0526 | Ascomycota | Chaetothyriales | Herpotrichiellaceae | Exophiala_quercina | 5 | 8 |
| OTU0527 | Ascomycota | Helotiales | unidentified | Helotiales_sp | 12 | 7 |
| OTU0528 | Ascomycota | Pleosporales | Parapyrenochaetaceae | Parapyrenochaeta_sp | 1 | 1 |
| OTU0529 | Basidiomycota | Hymenochaetales | Schizoporaceae | Oxyporus_corticola | 1 | 2 |
| OTU0530 | Ascomycota | Chaetothyriales | Herpotrichiellaceae | Cladophialophora_sp | 1 | 2 |
| OTU0531 | Ascomycota | Xylariales | unidentified | Xylariales_sp | 2 | 1 |
| OTU0534 | Basidiomycota | Auriculariales | Hyaloriaceae | Stypella_grilletii | 6 |  |
| OTU0535 | Ascomycota | Pleosporales | unidentified | Pleosporales_sp | 2 | 11 |
| OTU0536 | Basidiomycota | Corticiales | Corticiaceae | Corticiaceae_sp | 3 |  |
| OTU0537 | Basidiomycota | Tremellales | Bulleribasidiaceae | Vishniacozyma_victoriae | 6 | 8 |
| OTU0538 | Ascomycota | Sordariales | Sordariales_fam_Incertae_sedis | Cordana_sp | 1 | 2 |
| OTU0539 | Ascomycota | Chaetothyriales | Herpotrichiellaceae | Capronia_camelliae-yunnanensis | 3 | 3 |
| OTU0540 | Ascomycota | Pleosporales | Cucurbitariaceae | Neocucurbitaria_juglandicola | 17 | 14 |
| OTU0541 | Basidiomycota | Polyporales | Hyphodermataceae | Hyphoderma_setigerum | 5 |  |
| OTU0542 | Ascomycota | Phaeomoniellales | Phaeomoniellaceae | Nothophaeomoniella_ekebergiae | 2 | 5 |
| OTU0543 | Basidiomycota | Cystobasidiales | Cystobasidiaceae | Occultifur_sp | 19 | 15 |
| OTU0544 | Basidiomycota | Atractiellales | Phleogenaceae | Helicogloea_pellucida | 11 | 9 |
| OTU0545 | Ascomycota | Helotiales | Mollisiaceae | Phialocephala_lagerbergii | 4 |  |
| OTU0546 | Basidiomycota | Auriculariales | Auriculariales_fam_Incertae_sedis | Oliveonia_sp | 4 | 1 |
| OTU0547 | Ascomycota | Chaetothyriales | Herpotrichiellaceae | Herpotrichiellaceae_sp | 4 | 2 |
| OTU0548 | Ascomycota | Pleosporales | Cucurbitariaceae | Parafenestella_austriaca | 12 | 10 |
| OTU0550 | Ascomycota | Saccharomycetales | Metschnikowiaceae | Metschnikowia_sp |  | 2 |
| OTU0551 | Basidiomycota | Polyporales | Polyporales_fam_Incertae_sedis | Pycnoporellus_fulgens | 2 |  |
| OTU0552 | Ascomycota | Eurotiales | Aspergillaceae | Penicillium_sp | 20 | 10 |
| OTU0553 | Basidiomycota | Tremellales | unidentified | Tremellales_sp | 4 | 4 |
| OTU0554 | Ascomycota | Chaetothyriales | Herpotrichiellaceae | Herpotrichiellaceae_sp | 7 | 8 |
| OTU0555 | Basidiomycota | Tremellales | Carcinomycetaceae | Carcinomycetaceae_sp | 2 | 5 |
| OTU0556 | Ascomycota | Hypocreales | Hypocreaceae | Trichoderma_stellatum | 8 | 5 |
| OTU0557 | Ascomycota | Saccharomycetales | Metschnikowiaceae | Metschnikowia_continentalis | 2 | 1 |
| OTU0558 | Ascomycota | Ophiostomatales | Ophiostomataceae | Ophiostoma_sparsum | 2 |  |
| OTU0559 | Ascomycota | Eurotiales | Aspergillaceae | Rasamsonia_oblata | 1 |  |
| OTU0560 | Ascomycota | Chaetosphaeriales | Chaetosphaeriales_fam_Incertae_sedis | Dendrophoma_sp |  | 2 |
| OTU0561 | Ascomycota | Xylariales | Hypoxylaceae | Hypoxylon_sp | 6 | 1 |
| OTU0562 | Ascomycota | Mycocaliciales | Mycocaliciaceae | Mycocaliciaceae_sp | 6 | 5 |
| OTU0564 | Ascomycota | Xylariales | Diatrypaceae | Diatrypella_favacea | 1 |  |
| OTU0565 | Ascomycota | Chaetothyriales | Herpotrichiellaceae | Fonsecaea_sp | 13 | 6 |
| OTU0566 | Basidiomycota | Tremellales | unidentified | Tremellales_sp | 12 | 16 |
| OTU0567 | Basidiomycota | Hymenochaetales | Schizoporaceae | Oxyporus_corticola | 1 | 2 |
| OTU0568 | Basidiomycota | Polyporales | Phanerochaetaceae | Bjerkandera_adusta | 1 |  |
| OTU0569 | Ascomycota | Annulatascales | Annulatascaceae | Torrentispora_calembola | 3 |  |
| OTU0570 | Ascomycota | Saccharomycetales | Trichomonascaceae | Blastobotrys_sp |  | 1 |
| OTU0572 | Ascomycota | Chaetothyriales | unidentified | Chaetothyriales_sp | 15 | 18 |
| OTU0574 | Basidiomycota | Hymenochaetales | Schizoporaceae | Xylodon_chinensis | 2 |  |
| OTU0575 | Ascomycota | Pleosporales | unidentified | Pleosporales_sp | 1 | 1 |
| OTU0576 | Ascomycota | Calosphaeriales | Calosphaeriaceae | Flabellascus_tenuirostris | 1 | 1 |
| OTU0579 | Basidiomycota | Corticiales | Corticiaceae | Corticiaceae_sp | 4 |  |
| OTU0581 | Basidiomycota | Polyporales | Meruliaceae | Phlebia_tuberculata | 3 | 1 |
| OTU0582 | Basidiomycota | Filobasidiales | Piskurozymaceae | Solicoccozyma_terricola | 7 | 4 |
| OTU0583 | Ascomycota | Helotiales | unidentified | Helotiales_sp | 4 |  |
| OTU0584 | Basidiomycota | Auriculariales | unidentified | Auriculariales_sp | 1 | 1 |
| OTU0585 | Ascomycota | Xenospadicoidales | Xenospadicoidaceae | Pseudodiplococcium_sp | 3 | 3 |
| OTU0586 | Ascomycota | Xenospadicoidales | Xenospadicoidaceae | Pseudodiplococcium_sp | 5 | 2 |
| OTU0587 | Basidiomycota | Polyporales | Incrustoporiaceae | Skeletocutis_kuehneri | 2 |  |
| OTU0588 | Ascomycota | Chaetothyriales | Herpotrichiellaceae | Capronia_sp | 4 | 5 |
| OTU0590 | Basidiomycota | Agaricales | Mycenaceae | Mycena_crocata | 4 | 1 |
| OTU0591 | Ascomycota | Chaetothyriales | Herpotrichiellaceae | Capronia_pulcherrima | 18 | 9 |
| OTU0592 | unidentified | unidentified | unidentified | Fungi_sp |  | 8 |
| OTU0594 | Mucoromycota | Mucorales | Mucoraceae | Mucor_hiemalis | 9 |  |
| OTU0595 | Basidiomycota | Auriculariales | Auriculariaceae | Auricularia_mesenterica | 1 |  |
| OTU0596 | Ascomycota | Pleosporales | unidentified | Pleosporales_sp | 5 | 5 |
| OTU0598 | Ascomycota | Helotiales | Helotiaceae | Scytalidium_lignicola | 2 | 5 |
| OTU0599 | Ascomycota | unidentified | unidentified | Lecanoromycetes_sp | 1 | 7 |
| OTU0600 | Basidiomycota | Auriculariales | Exidiaceae | Exidia_sp | 1 |  |
| OTU0603 | Basidiomycota | Polyporales | Meruliaceae | Phlebia_tuberculata | 1 | 1 |
| OTU0604 | Ascomycota | Saccharomycetales | Debaryomycetaceae | Meyerozyma_amylolytica |  | 8 |
| OTU0605 | Ascomycota | Chaetothyriales | Herpotrichiellaceae | Herpotrichiellaceae_sp | 13 | 2 |
| OTU0606 | Basidiomycota | Polyporales | Incrustoporiaceae | Skeletocutis_kuehneri | 2 |  |
| OTU0608 | Ascomycota | Chaetothyriales | Herpotrichiellaceae | Fonsecaea_sp | 7 | 3 |
| OTU0609 | Ascomycota | Hypocreales | Hypocreales_fam_Incertae_sedis | Natantiella_ligneola | 10 | 3 |
| OTU0612 | Basidiomycota | Cantharellales | Hydnaceae | Sistotrema_raduloides | 1 |  |
| OTU0615 | Ascomycota | Chaetosphaeriales | Chaetosphaeriaceae | Chaetosphaeria_sp | 12 | 2 |
| OTU0616 | Basidiomycota | Sebacinales | Sebacinaceae | Sebacinaceae_sp | 7 | 4 |
| OTU0617 | Basidiomycota | Agaricales | Mycenaceae | Mycena_galericulata | 4 | 1 |
| OTU0618 | Basidiomycota | Cantharellales | Ceratobasidiaceae | Thanatephorus_ochraceus | 13 | 4 |
| OTU0620 | Ascomycota | Chaetothyriales | Herpotrichiellaceae | Rhinocladiella_quercus | 4 | 9 |
| OTU0621 | Basidiomycota | Agaricales | Crepidotaceae | Simocybe_haustellaris |  | 2 |
| OTU0625 | unidentified | unidentified | unidentified | Fungi_sp | 5 | 6 |
| OTU0627 | Basidiomycota | Leucosporidiales | Leucosporidiaceae | Leucosporidium_creatinivorum | 7 | 2 |
| OTU0641 | Basidiomycota | Agaricales | Strophariaceae | Pholiota_adiposa | 7 | 1 |
| OTU0643 | Ascomycota | Chaetothyriales | Herpotrichiellaceae | Capronia_coronata | 8 | 13 |
| OTU0651 | Basidiomycota | Cantharellales | unidentified | Cantharellales_sp | 6 | 2 |
| OTU0653 | Basidiomycota | Microbotryomycetes_ord_Incertae_sedis | Microbotryomycetes_fam_Incertae_sedis | Curvibasidium_cygneicollum | 2 | 3 |
| OTU0654 | Basidiomycota | Agaricales | Mycenaceae | Mycena_galericulata | 1 |  |
| OTU0686 | Basidiomycota | Atractiellales | Phleogenaceae | Helicogloea_insularis | 11 | 3 |
| OTU0691 | Basidiomycota | Cystofilobasidiales | Mrakiaceae | Mrakia_frigida | 4 | 2 |
| OTU0697 | Basidiomycota | Agaricales | Agaricales_fam_Incertae_sedis | Chondrostereum_purpureum | 6 |  |
| OTU0765 | Ascomycota | Chaetothyriales | Herpotrichiellaceae | Exophiala_sp | 9 | 14 |
| OTU0822 | Basidiomycota | Russulales | Auriscalpiaceae | Lentinellus_ursinus |  | 1 |


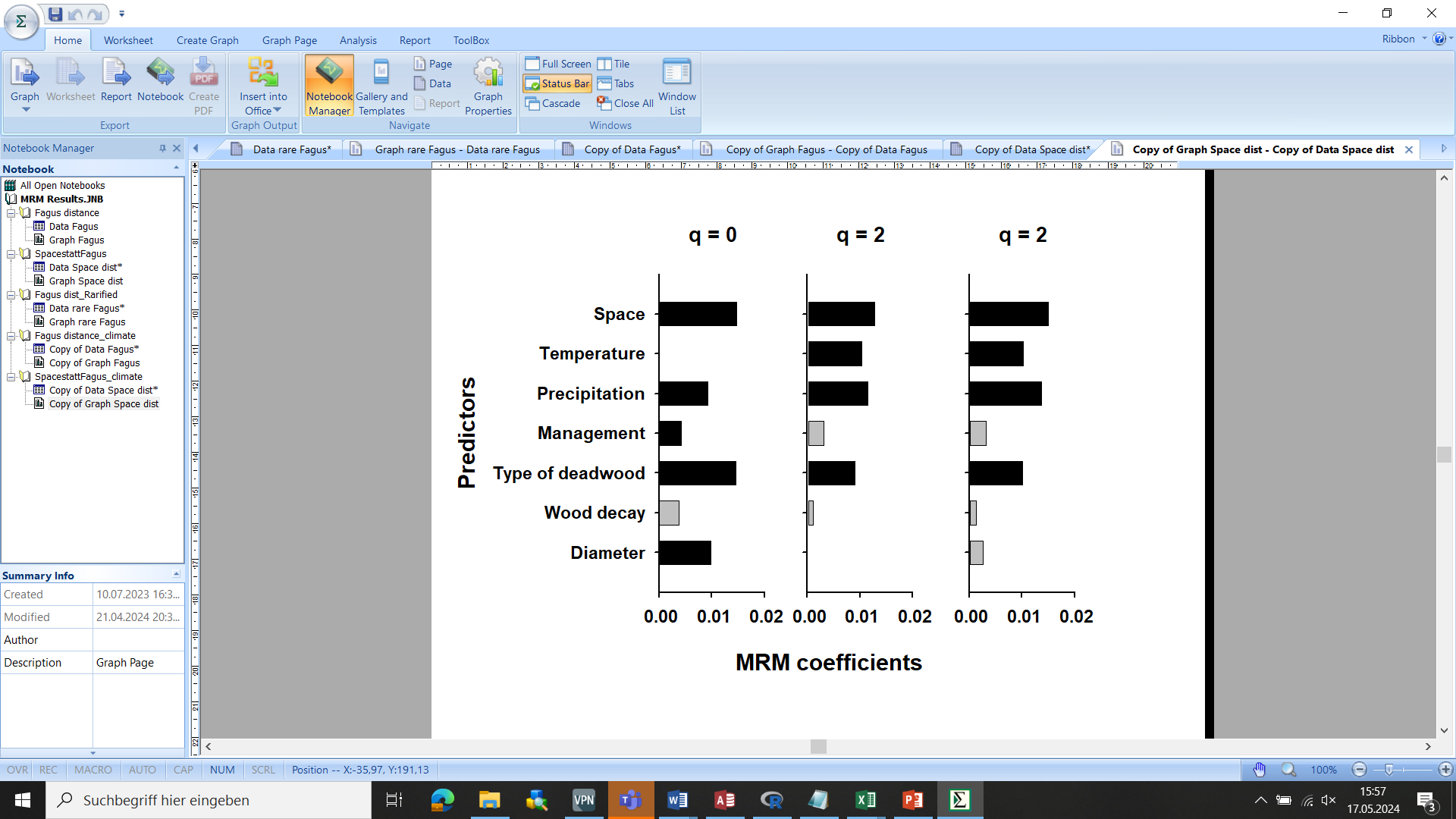


**Fig. S1:** Multiple regression on distance matrices (MRM) coefficients of the predictors of the composition of fungi in 215 deadwood objects located in 18 beech forests as in Figure 1, but using spatial distance instead of *Fagus* species as predictor. Black bars indicate significance in MRM p < 0.05. Results are shown only for rare (q=0), common (q=1) and dominant (q=2) fungi species. *Management* compared production with old-growth forest stands.


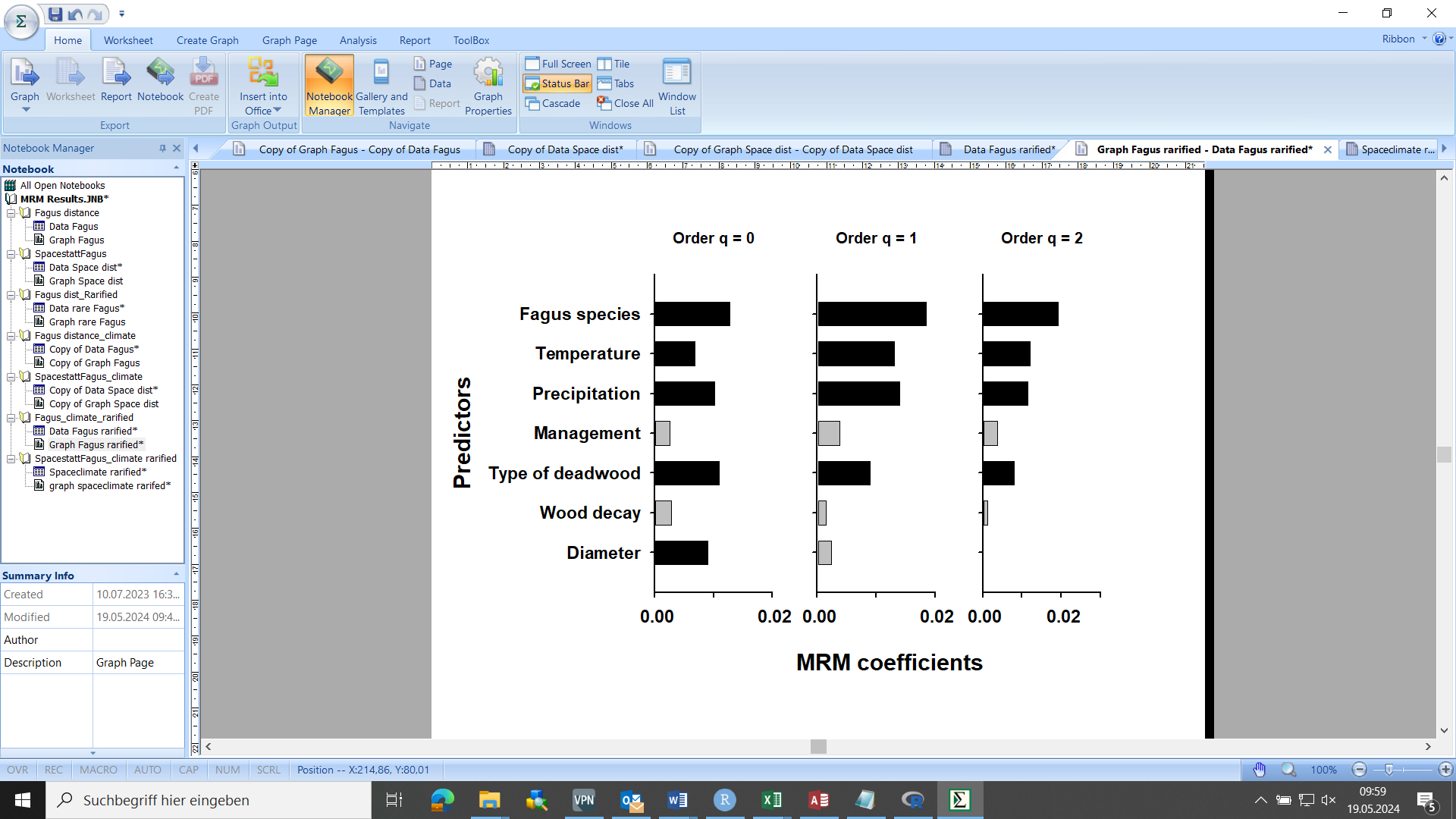


**Fig. S2:** Multiple regression on distance matrices (MRM) coefficients of the predictors of the composition of fungi in 215 deadwood objects located in 18 beech forests as in Figure 1, but based on rarified communities. Black bars indicate significance in MRM p < 0.05. Results are shown only for rare (q=0), common (q=1) and dominant (q=2) fungi species. *Management* compared production with old-growth forest stands, and *Fagus* *species* compared *F*. *sylvatica* with *F*. *orientalis*.

**Tab. S2**: Results of a generalized linear mixed model with negative binomial distribution and forest as random factor for species per object identified by metabarcoding, observed is based on all species after removing single hits, the rarified richness is based on the community after rarifying samples to 990 hits; * p<0.05.

|  | Observed | Rarified |
| --- | --- | --- |
|  | z-value / p value | z-value / p value |
| Temperature | 1.49 | 1.21 |
| Precipitation | 0.21 | 0.43 |
| Decay stage | 2.22* | 2.21* |
| Diameter | -0.84 | -1.43 |
| Object Standing *vers* Log | 0.38 | 0.46 |
| Object Stump *vers* Log | 0.27 | 1.51 |
| Fagus species | -0.32 | -0.16 |
| Management: Old-growth | 0.04 | 0.21 |
